# High-grade gliomas where resources define survival: a systematic review and meta-analysis of outcomes, surgical access, and molecular diagnostic gaps in low- and middle-income countries

**DOI:** 10.3389/fonc.2026.1909193

**Published:** 2026-09-30

**Authors:** Carlos Castillo Rangel, Gervith Reyes Soto, Edinson David Berrio Perea, José De Jesús Gutiérrez-Baños, Eduardo Diaz Juarez, Bayron Salazar, Vishal K. Chavda, Ismael Antonio Peralta Baez, Renat Nurmukhametov, David Cuevas, Lizardi de la Cruz, Andreina Rosario Rosario, Bary Bigay, Abuzer Gungor, Manuel De Jesus Encarnacion Ramirez

**Affiliations:** 1 Department of Neurosurgery, Servicio of the 1ro de Octubre Hospital of the Instituto de Seguridad y Servicios Sociales de los Trabajadores del Estado, Instituto Politécnico Nacional, México, Mexico; 2 Neurosurgical Oncology, Mexico’s National Institute of Cancer, Tlalpan, Mexico; 3 Department of Neurosurgery, Centro Medico ISSEMYM Toluca, Toluca, Mexico; 4 Department of Neurosurgery, National University of Mexico Hospital General, Durango, Mexico; 5 Department of Neurosurgery, Hospital Militar de Quito, Quito, Ecuador; 6 Department of Neurology, Neurosurgery & Neurogenetics, ChromoMED Institute, Santo Domingo, Dominican Republic; 7 Department of Neurosurgery, Hospital Regional Alejandro Cabral, San Juan de la Maguana, Dominican Republic; 8 Department of Neurosurgery, Russian People’s Friendship University, Moscow, Russia; 9 Intensive Care Unit, Hospital Traumatológico Dr. Ney Arias Lora, Santo Domingo, Dominican Republic; 10 Faculty of Medicine, Autonomous University of Santo Domingo, Santo Domingo, Dominican Republic; 11 ChromoMED Institute, Santo Domingo, Dominican Republic; 12 Istinye Universitesi, İstanbul, Türkiye; 13 Department of Neurosurgery, Hospital traumatologico de Azua, Azua, Dominican Republic; 14 Assistant of the department of Human Anatomy and Histology of the Institute of Clinical Medicine named after N.V. Sklifosovskiy, Moscow, Russia; 15 Departament of Neurology, Neurosurgery and Neurogenetic ChromoMED Institute, Santo Domingo, Dominican Republic; 16 Hospital Traumatológico Dr. Ney Arias Lora, Santo Domingo, Dominican Republic

**Keywords:** extent of resection, glioblastoma, global neurosurgery, health-system disparities, high-grade glioma, IDH mutation, LMICs, low- and middle-income countries

## Abstract

**Background:**

High-grade gliomas remain among the most aggressive tumors in neuro-oncology. Although prognosis is strongly influenced by tumor biology, survival in low- and middle-income countries is also shaped by health-system factors, including delayed diagnosis, limited surgical infrastructure, incomplete adjuvant therapy, and restricted molecular diagnostics. The implementation of the 2021 World Health Organization Classification of Central Nervous System Tumors has further exposed diagnostic inequities, as integrated histomolecular classification depends on molecular tools that are not uniformly available in resource-limited settings.

**Objective:**

This systematic review and meta-analysis aimed to synthesize contemporary evidence on survival outcomes, surgical access, extent of resection, adjuvant treatment delivery, and molecular diagnostic gaps among patients with high-grade gliomas treated in low- and middle-income countries.

**Methods:**

A systematic search was conducted following PRISMA 2020 recommendations. MEDLINE/PubMed, Embase, Scopus, Web of Science, Cochrane CENTRAL, Global Index Medicus, LILACS, African Index Medicus, IndMED, and Google Scholar were searched for studies published between January 2016 and May 2026. Eligible studies included patients with histologically or molecularly confirmed WHO grade 3 or grade 4 gliomas treated in low- or middle-income countries and reporting survival, surgical-access, adjuvant-treatment, or molecular-diagnostic outcomes. The protocol was registered in PROSPERO as CRD420261398058.

**Results:**

A total of 30 independent studies met the predefined eligibility criteria and were included in the qualitative synthesis. Twenty-four studies contributed overall survival data, 18 reported surgical-access outcomes, 21 evaluated adjuvant-treatment delivery, and 12 examined molecular-diagnostic availability. Most included investigations were retrospective institutional cohorts, although registry-based studies, randomized clinical trials, and molecular implementation studies were also identified. Survival outcomes varied substantially across healthcare settings and were consistently influenced by delayed diagnosis, limited access to maximal safe resection, incomplete radiotherapy, restricted temozolomide availability, treatment abandonment, and insufficient molecular characterization.

**Conclusions:**

Survival in high-grade gliomas in low- and middle-income countries is not determined by tumor biology alone. It is shaped by sequential “survival gates”: timely diagnosis, access to maximal safe resection, completion of radiotherapy and temozolomide-based chemotherapy, molecular classification, and longitudinal follow-up. Future strategies should move beyond simple guideline translation and focus on scalable neuro-oncology pathways that improve surgical capacity, adjuvant-treatment continuity, and access to essential molecular diagnostics.

**Systematic review registration:**

https://www.crd.york.ac.uk/PROSPERO/view/CRD420261398058, identifier CRD420261398058.

## Introduction

Unfortunately, despite their aggressiveness and early recurrences, the prognosis of high-grade gliomas is increasingly determined by the health system within which the tumor is diagnosed and treated. Thus, under adequately resourced conditions, current treatment of high-grade glioma is not based solely on identifying the corresponding anaplastic/glioblastomatous morphology. It should involve a complex of measures, including high-quality neuro-imaging, safe and appropriate cytoreductive surgery, neuropathological evaluation, immunohistochemistry, molecular diagnostics, radiotherapy, alkylating chemotherapy, and monitoring. The introduction of the WHO Classification of Central Nervous System Tumors (5th Edition), which emphasized the role of molecular biomarkers in diagnosing, prognosticating, and planning therapy, was especially important in regard to high-grade glioma and specifically diffuse adult-type gliomas, such as glioblastoma, IDH-wildtype; astrocytoma, IDH-mutant; and oligodendroglioma, IDH-mutant and 1p/19q-codeleted (1). Consequently, currently, a diagnosis of “high-grade glioma” without further characterization by molecular biomarkers might be insufficient for several reasons and even misleading because different subtypes of high-grade glioma are biologically diverse (1–3).

However, for many low- and middle-income countries, the progress in molecular classification of brain tumors has become a new challenge. Despite the obvious benefits of molecular diagnostics and its importance for differential diagnosis and personalized treatment, this approach has increased the gap between recommended guidelines for managing high-grade glioma and real clinical practices. The key elements of the current therapeutic architecture include maximal safe resection of the tumor tissue, postoperative radiotherapy, chemotherapy with temozolomide (where needed), and certain molecular biomarkers (such as IDH status, MGMT promoter methylation, TERT promoter mutation, EGFR amplification, chromosome +7/−10 signature, ATRX loss, CDKN2A/B homozygous deletion, and 1p/19q codeletion) (2, 3). Importantly, the use of the aforementioned markers goes beyond academic considerations. For example, their status affects the decision concerning the need for and type of surgical intervention as well as participation in certain clinical trials.

The global burden of brain and central nervous system tumors also reveals another problem that must be addressed. According to recent epidemiologic estimates, there are significant discrepancies in the incidence, mortality rates, and burden of brain/CNS tumors between high-income countries and LMICs, suggesting a trend towards underreporting and underdiagnosis, and even increasing mortality rate from the disease (4). Although in high-income countries the incidence and prevalence of certain types of brain/CNS cancer can be reliably estimated with the help of population-based cancer registries, in some LMICs, reliable data on the burden of various CNS malignancies are absent due to the lack of cancer registries and comprehensive databases on neuro-oncology (5).

Surgery is the first point in the therapy of a high-grade glioma, where the lack of resources has a direct impact on the patients’ survival. Namely, it has been repeatedly shown that the extent of resection has a significant positive effect on overall and progression-free survival among patients who undergo maximal safe resection (6). However, to achieve the desired outcome, not only a skilled neurosurgeon is required. Adequate diagnostics, access to MRI equipment, an equipped operating room with anesthesia assistance, adequate intensive care capacity and availability of blood transfusions, a microscope, neuronavigation/fMRI-based functional mapping, a perioperative team, and facilities for managing possible complications are necessary. Unfortunately, in many LMICs, resource constraints lead to delayed consultations with specialists, difficulties accessing MRI, limited availability of surgical facilities and skilled neurosurgeons, and inadequate intensive care capabilities (7, 8). As a result, the possibility of performing surgery and obtaining good results is limited, and patients might undergo biopsy, subtotal resection, or delayed and inadequate surgery. Moreover, due to limited hospital capacity, it is difficult to operate on many patients with high-grade glioma.

Adjuvant therapy is the second factor that can affect disease treatment and patient survival. High-grade glioma therapy is highly sensitive to timing; consequently, delayed initiation of radiotherapy, chemotherapy, and follow-up imaging might lead to reduced efficacy of the treatment and poor results (9). According to LMIC-focused reviews, access to the aforementioned factors can be significantly hindered by limited radiotherapy capacity, restricted availability of necessary medication and equipment, inability to afford the costs of therapy, the complexity of the referral pathway, lack of capacity to treat patients locally, high costs of travel, hospitalization, and lost income, etc. (9). As a consequence, patients can lose their chances to receive maximum benefit from the surgery. This problem is especially acute for glioblastoma and other diffuse grade 4 gliomas, where months can determine a patient’s lifespan. Therefore, the survival rate cannot be discussed without taking into account both the quality of the surgery and the availability of adjuvant therapy.

Finally, another aspect of the problem under discussion lies in the fact that proper classification of brain tumors is crucial to provide a correct diagnosis and proper therapy. According to the current WHO Classification of CNS Tumors, accurate determination of IDH, MGMT, ATRX, and 1p/19q status is indispensable for classifying most gliomas. Recent research from Pakistan demonstrated that the implementation of the WHO 2021 criteria and the inability to test molecular characteristics resulted in about 40% of cases being unable to be reliably classified according to the latest recommendations. Similar results were obtained in a high-volume Latin American neurosurgical center, where the WHO 2021 classification led to an increased proportion of not otherwise specified gliomas and high-grade/low-grade glioma NOS due to a lack of molecular information (10, 11). Consequently, instead of increasing precision, WHO 2021 might lead to more uncertainties in diagnosis and treatment.

As mentioned before, despite the increasing number of publications on high-grade glioma therapy in LMICs, most suffer from significant biases. Namely, many studies are retrospective, single-center studies with incomplete molecular data, unclear descriptions of the extent of resection performed, incomplete information about follow-up procedures (radiotherapy, chemotherapy), short observation periods, and insufficient follow-up (11–17). The survival rates are presented without separation of histological glioblastoma from WHO 2021-defined glioblastoma, IDH-wildtype and without mentioning IDH/MGMT testing. As a result, the data on the effectiveness of the current therapeutic strategy are difficult to evaluate in terms of its real successes and failures. Without taking into account the problems of adjuvant treatment, surgical access, and diagnostics, survival cannot be considered an objective characteristic of the disease.

Consequently, a resource-sensitive approach to analyzing survival rates in high-grade glioma and examining barriers in LMICs is essential for a better understanding of the problems encountered by clinicians and how to tackle them. For this purpose, the following systematic review and meta-analysis of contemporary literature were undertaken.

## Materials and methods

### Study design and reporting framework

This systematic review and endpoint-specific meta-analysis was conducted in accordance with the Preferred Reporting Items for Systematic Reviews and Meta-Analyses 2020 statement. The methodological approach was based on the Cochrane Handbook for Systematic Reviews of Interventions, adapted for observational cohorts, registry-based studies, non-randomized comparative studies, and resource-stratified health systems research. The review protocol was registered in the International Prospective Register of Systematic Reviews under the registration number CRD420261398058.

Thirty independent studies were included in the qualitative synthesis. Because this study was based exclusively on previously published aggregate data and did not involve individual patient-level information, institutional review board approval and informed consent were not required.

### Review question and conceptual framework

The review evaluated the management and outcomes of patients with high-grade gliomas treated in low- and middle-income countries (LMICs). The primary outcome was overall survival. Secondary outcomes included extent of resection, postoperative complications, access to radiotherapy and temozolomide, and molecular diagnostic availability, particularly IDH and MGMT testing. The review was conceptually based on the premise that survival in LMICs is influenced by sequential resource-dependent steps throughout the treatment pathway.

### Eligibility criteria

Studies were eligible if they met all of the following criteria: original clinical research; publication between January 1, 2016, and May 10, 2026; inclusion of patients with high-grade glioma; treatment or diagnosis in at least one low- or middle-income country; and reporting of at least one outcome related to survival, surgical access, adjuvant treatment, postoperative complications, or molecular diagnostic availability.

Eligible study designs included retrospective cohorts, prospective cohorts, institutional series, cancer registry studies, population-based studies, randomized or non-randomized comparative studies, and molecular diagnostic implementation studies when clinical or diagnostic outcomes were extractable. Randomized trials were included only when they enrolled patients treated in LMIC settings or provided separately extractable LMIC data.

Studies were excluded if they were case reports, narrative reviews, systematic reviews, meta-analyses, editorials, expert opinions, preclinical studies, technical reports without clinical outcomes, or case series including fewer than 10 patients with high-grade gliomas. Studies conducted exclusively in high-income countries were excluded unless they reported an independently extractable LMIC subgroup. Mixed tumor cohorts were included only when high-grade glioma data could be extracted separately. Conference abstracts were used only to identify potentially eligible full-text publications; they were not included in quantitative synthesis unless a peer-reviewed full text with sufficient extractable data was available.

Because glioma classification changed substantially across the study period, cohorts using older WHO classifications were not automatically excluded. Instead, they were categorized according to diagnostic era and degree of molecular integration. When possible, diagnoses were mapped descriptively to contemporary WHO CNS5 terminology; however, histology-only cohorts were not pooled indiscriminately with fully molecularly integrated cohorts.

### Information sources and search strategy

A systematic search was conducted in MEDLINE/PubMed, Embase, Scopus, Web of Science Core Collection, Cochrane CENTRAL, Global Index Medicus, LILACS, African Index Medicus, Index Medicus for the Eastern Mediterranean Region, IndMED, and Google Scholar. The final search was performed on May 10, 2026. The reference lists of eligible articles and relevant reviews were manually screened to identify additional studies.

The search strategy combined terms related to high-grade glioma, glioblastoma, LMIC status, survival, surgery, adjuvant therapy, and molecular diagnosis. The PubMed strategy was adapted for each database according to its syntax and indexing structure. The core search pattern was:

(“high-grade glioma” OR “high grade glioma” OR glioblastoma OR gliosarcoma OR “anaplastic astrocytoma” OR “WHO grade III glioma” OR “WHO grade IV glioma” OR “diffuse glioma” OR “diffuse midline glioma”) AND (“low-income country” OR “middle-income country” OR LMIC OR “resource-limited” OR “resource constrained” OR “developing country” OR “global neurosurgery” OR Africa OR Asia OR Latin America OR Caribbean OR “Eastern Europe” OR “Sub-Saharan Africa” OR India OR Pakistan OR Bangladesh OR Nigeria OR Kenya OR Ethiopia OR Egypt OR Morocco OR Tunisia OR Brazil OR Mexico OR Colombia OR Peru OR Dominican Republic OR Indonesia OR Philippines OR Vietnam OR Iran OR Iraq OR Ukraine OR Ghana OR Tanzania OR Uganda) AND (survival OR outcome OR mortality OR “overall survival” OR “progression-free survival” OR surgery OR resection OR biopsy OR radiotherapy OR temozolomide OR chemotherapy OR IDH OR MGMT OR molecular OR “molecular diagnosis” OR “WHO 2021”).

No language restriction was applied. Articles in English, Spanish, Portuguese, French, and Russian were assessed directly by members of the review team. Articles in other languages were screened using translated titles and abstracts and were considered eligible if sufficient information could be reliably extracted.

For Google Scholar, the first 200 results sorted by relevance were screened using the same core concepts, and additional records were considered only when they clearly addressed high-grade glioma outcomes, treatment access, or molecular diagnostic barriers in LMICs.

### Study selection

All records were exported into a reference manager, and duplicate records were removed before screening. Titles and abstracts were screened independently by two reviewers. Records considered potentially eligible by either reviewer proceeded to full-text assessment.

Full-text articles were then reviewed independently by two reviewers according to the predefined eligibility criteria. Disagreements were resolved through discussion. If consensus could not be reached, a third reviewer adjudicated the decision. Reasons for exclusion at the full-text stage were recorded and summarized in the PRISMA flow diagram.

When multiple reports appeared to derive from the same institution, overlapping study period, or same patient cohort, the article with the largest sample size, longest follow-up, most complete molecular data, or most detailed outcome reporting was selected for primary extraction. Companion reports were used only for supplementary variables when patient duplication did not affect the outcome being analyzed.

### Data extraction

Data were extracted independently by two reviewers using a standardized extraction form. Collected variables included study characteristics, patient demographics, tumor subtype, WHO classification, surgical treatment, extent of resection, adjuvant therapy, molecular diagnostic availability (including IDH and MGMT testing), and survival outcomes. Additional information regarding postoperative complications and follow-up was extracted whenever available.

### Outcome definitions

Overall survival was the primary endpoint and was defined according to each included study. Progression-free survival, extent of resection, adjuvant therapy, and molecular diagnostic availability were analyzed using the definitions reported by the original studies.

### Risk of bias assessment

Risk of bias was assessed independently by two reviewers. Non-randomized comparative studies evaluating treatment effects were assessed using ROBINS-I. Observational cohort and registry-based studies were evaluated with the Newcastle-Ottawa Scale, considering representativeness, comparability, outcome ascertainment, and follow-up adequacy. Uncontrolled case series were assessed using the Joanna Briggs Institute critical appraisal tool for case series.

Particular attention was given to selection bias, immortal time bias, incomplete follow-up, inconsistent survival origin, unadjusted confounding, unclear extent-of-resection definitions, incomplete adjuvant-treatment reporting, and lack of molecular integration. Disagreements in risk-of-bias assessment were resolved by consensus or by adjudication from a third reviewer. Risk-of-bias judgments were incorporated into sensitivity analyses and into the certainty-of-evidence assessment.

### Data synthesis and statistical analysis

A structured narrative synthesis was conducted for all included studies. Studies were grouped according to geographic region, World Bank income category, tumor type, diagnostic era, study design, treatment pathway, and degree of molecular diagnostic completeness.

Quantitative synthesis was performed only when at least three studies reported the same outcome with sufficiently comparable definitions and extractable denominators. Because of substantial clinical, molecular, and methodological heterogeneity across adult glioblastoma, mixed adult high-grade glioma, pediatric high-grade glioma, diffuse midline glioma, and histology-only cohorts, no overall pooled estimate was generated for all high-grade gliomas.

Endpoint-specific random-effects meta-analyses were performed for outcomes with adequate methodological comparability, including overall survival, biopsy-only rate, gross-total or near-total resection, radiotherapy receipt, temozolomide use, chemoradiotherapy completion, IDH testing, MGMT testing, and molecularly incomplete (NOS/NEC) diagnoses. Studies with unclear denominators, incompatible outcome definitions, or non-extractable subgroups were excluded from quantitative pooling but were retained in the narrative synthesis.

Heterogeneity was assessed using Cochran’s Q statistic, I², and between-study variance, with I² interpreted cautiously given the expected clinical heterogeneity among studies. Subgroup analyses were performed whenever sufficient data were available according to income category, geographic region, tumor subtype, WHO classification era, adult versus pediatric population, availability of molecular diagnostics, and access to radiotherapy or temozolomide. Meta-regression was not performed because the available data were insufficient to support robust multivariable analyses across the predefined outcomes.

All statistical analyses were performed using R software (meta and metafor packages). Forest plots were generated for all pooled analyses, funnel plots were constructed when appropriate to explore publication bias, and statistical significance was defined as a two-sided *p* value < 0.05.

### Certainty of evidence

The certainty of evidence for each major outcome was assessed using the GRADE approach, considering risk of bias, inconsistency, indirectness, imprecision, and publication bias.

### Handling of missing data

Missing variables were treated as not reported. No statistical imputation was performed. When survival, treatment, or molecular data were unclear, corresponding authors were contacted when contact information was available. If no clarification was obtained, only extractable data with clear denominators were used in quantitative synthesis. Studies with incomplete denominators were retained in narrative synthesis when clinically relevant but were excluded from pooled analyses.

## Results

### Study selection

The systematic search identified 948 records through electronic database searching and additional citation tracking. After the removal of 210 duplicate records, 738 unique citations remained for title and abstract screening. Of these, 679 records were excluded because they were unrelated to high-grade gliomas, were conducted exclusively in high-income countries without separately extractable LMIC data, represented reviews, editorials, conference abstracts without eligible full-text publications, or failed to report clinically relevant outcomes.

A total of 59 full-text articles were sought for eligibility assessment. Four reports could not be retrieved despite institutional library access and manual searching, leaving 55 full-text articles for detailed review. After full-text evaluation, 25 studies were excluded because they did not satisfy the predefined eligibility criteria, including ineligible study design, absence of extractable LMIC-specific data, duplicate patient populations, insufficient outcome reporting, or mixed tumor cohorts without separable high-grade glioma data.

Ultimately, 30 independent studies fulfilled all eligibility criteria and were included in the qualitative synthesis. Among these, 24 studies contributed data on overall survival, 18 provided extractable surgical-access and extent-of-resection outcomes, 21 reported adjuvant-treatment variables, and 12 contained sufficient molecular diagnostic data for endpoint-specific quantitative synthesis. The complete study selection process is summarized in **Figure 1**, which follows the PRISMA 2020 reporting framework.

**Figure 1:**
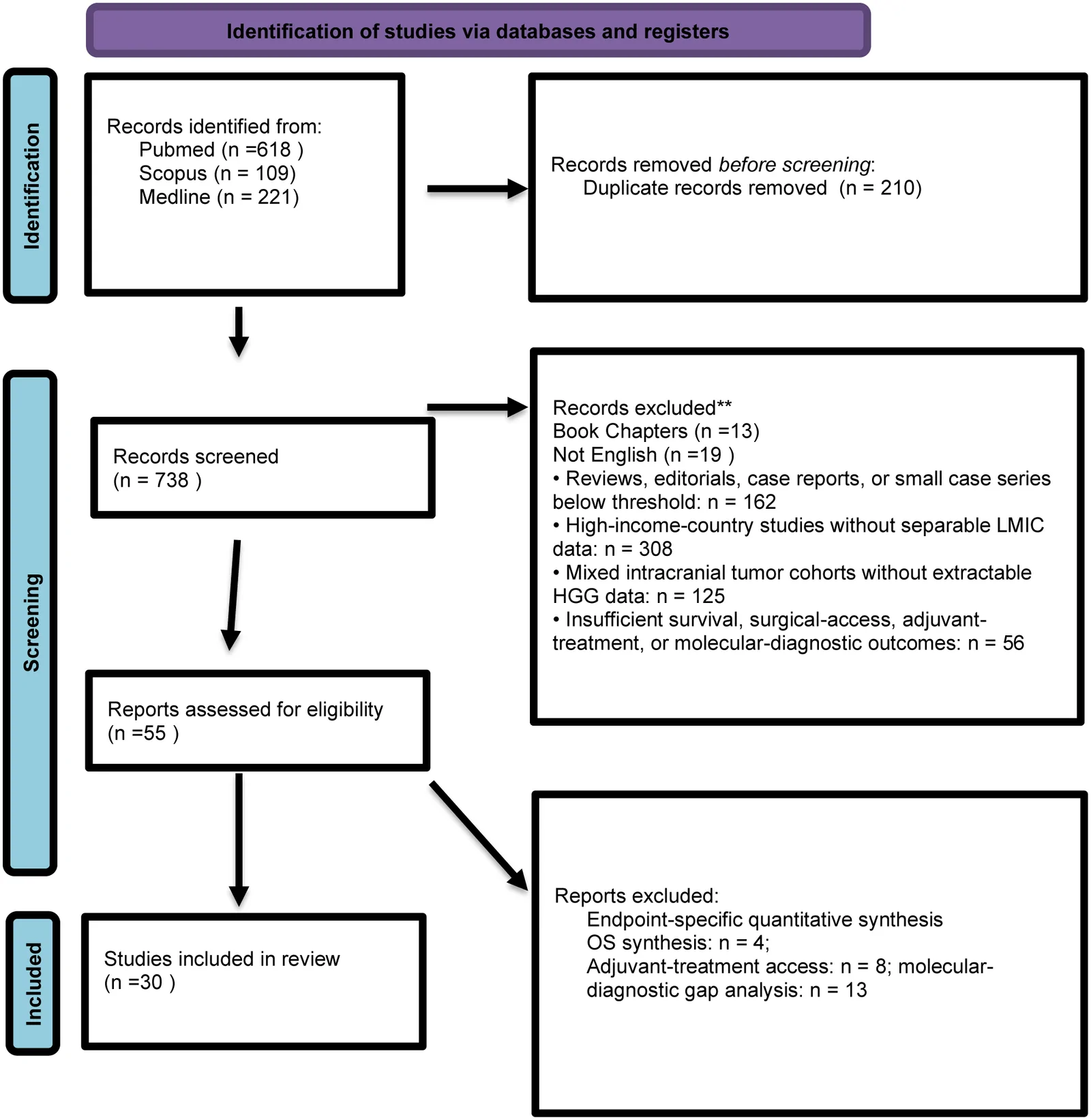
PRISMA 2020 flow diagram.

### Characteristics of included studies

The 30 included studies, published between 2016 and 2026, represented a geographically diverse LMIC evidence base spanning South Asia, Southeast Asia, East Asia, Latin America, North Africa, Sub-Saharan Africa, the Middle East, and Eastern Europe. Most investigations were retrospective institutional cohorts, although national registry studies, prospective cohorts, randomized controlled trials, quality-of-care audits, molecular implementation studies, and population-based analyses were also represented.

Glioblastoma constituted the predominant tumor type across the included literature, whereas several studies enrolled mixed cohorts of WHO grade 3 and grade 4 diffuse gliomas. Because diagnostic criteria evolved substantially following the publication of the WHO 2021 Classification of Central Nervous System Tumors, the included studies represented different diagnostic eras, ranging from purely histology-based classifications to fully integrated histomolecular diagnoses. Rather than excluding earlier cohorts, studies were stratified according to the degree of molecular integration to preserve the real-world representation of neuro-oncology practice across LMICs.

Most reports originated from tertiary referral hospitals with specialized neurosurgical services, potentially overestimating access to advanced imaging, maximal safe resection, adjuvant radiotherapy, temozolomide, and molecular diagnostics compared with national practice. Conversely, registry-based and public-sector studies provided broader insight into healthcare-system barriers, treatment abandonment, delayed diagnosis, and disparities in survival at the population level. This distinction was considered essential when interpreting pooled estimates because institutional cohorts frequently reflect the highest level of care available within each country rather than the average standard of care.

The principal characteristics of the 30 included studies are summarized in **Table 1**. Together, these studies provide a geographically diverse representation of neuro-oncology practice across LMICs, encompassing a broad spectrum of study designs, healthcare settings, treatment strategies, molecular diagnostic implementation, and survival outcomes. This descriptive overview provides the contextual framework for the endpoint-specific descriptive findings and quantitative syntheses presented in the following sections.

**Table 1:** Independent studies included in the qualitative synthesis (n = 30).

Study identification	Patients/sample size	Design and population	Treatment/surgical access variables	Molecular/technology variables	Main extractable outcomes	Synthesis domain/survival time
Hong et al., 2022 — Philippines (12)	48	Retrospective cohort; surgically managed glioblastoma patients in a tertiary government referral hospital	Surgery, extent of resection, adjuvant therapy access, socioeconomic status	Limited molecular reporting; public-sector resource constraints	Median OS; adjuvant-treatment receipt; socioeconomic predictors of survival	OS; surgical access; adjuvant access; socioeconomic determinants. Median OS: 7.6 months
Rozumenko et al., 2023 — Ukraine (13)	2,973	National Cancer Registry-based glioblastoma cohort	Standard-care compliance, surgery, RT, chemotherapy	National-level treatment availability; limited granular molecular data	OS; treatment-pattern compliance; national care gaps	OS; treatment compliance; health-system access. Survival time: reported; exact median/interval to verify in extraction sheet
Shah et al., 2024 — Pakistan (14)	285	Retrospective 10-year HGG cohort	Treatment patterns; survival by therapy	IDH, ATRX, 1p/19q; molecular epidemiology	OS; PFS; molecular survival correlates	OS; PFS; molecular subgroup analysis. Survival time: reported; exact median OS/PFS to verify in full text
Rehman et al., 2026 — Pakistan (15)	85	Young-adult HGG cohort	Treatment and survival outcomes in young adults	IDH, MGMT, ATRX; molecular profiling	Median OS; IDH-wildtype frequency; MGMT methylation in IDH-wildtype tumors	Molecular diagnostic access; young-adult HGG subgroup. Median OS: 16.5 months
Jilla et al., 2022 — India (16)	46	Retrospective tertiary-care glioblastoma cohort	Surgery, extent of excision, adjuvant therapy, TMZ completion	IDH/MGMT testing unavailable or limited	1-year OS; 2-year OS; prognostic factors	OS; adjuvant completion; molecular gap. Median OS: 8 months; 1-year OS: 36.9%; 2-year OS: 10.8%
Achari et al., 2022 — India (17)	106	Institutional glioblastoma quality-of-care audit	Surgery-to-adjuvant pathway; RT/TMZ process indicators	Quality-indicator framework; institutional care metrics	Compliance with quality indicators; treatment-delivery gaps	Health-system quality; Median OS: 20.0 months; median PFS: 11.0 months; median follow-up: 24 months
Mohammed et al., 2022 — India (18)	60	Retrospective GBM cohort treated with adjuvant chemoradiotherapy	Surgery, RT, chemotherapy	Conventional chemoradiotherapy pathway	Median OS; 1-year OS; 2-year OS; QoL	OS; adjuvant chemoradiotherapy. Median OS: 10 months; 1-year OS: 30%; 2-year OS: 6.7%
Dewangan et al., 2025 — India (19)	72	Retrospective HGG cohort treated with surgery and adjuvant therapy	Surgery, RT, chemotherapy	Standard clinicopathological variables	Median OS; prognostic survival factors	Median overall survival 15 months; median progression-free survival 8 months.
Bhandari et al., 2017 — India (20)	40	Comparative adjuvant TMZ-duration study in newly diagnosed GBM	Six versus 12 cycles of postoperative TMZ	Chemotherapy-duration comparison	OS by TMZ-cycle exposure	Adjuvant TMZ subgroup. Survival time: reported by TMZ-cycle group; exact group values to verify before pooling
Mallick et al., 2018 — India (21)	89 randomized; 83 analyzed	Phase II randomized trial in newly diagnosed GBM	Hypofractionated accelerated RT with TMZ	Resource-adapted RT/TMZ strategy	OS; PFS; toxicity; feasibility	RT strategy; adjuvant-treatment access. Survival time: reported; exact OS/PFS to verify before pooled RT analysis
Mallick et al., 2023 — India (22)	89 randomized; 83 analyzed	Updated phase II HART-GBM analysis	Hypofractionated accelerated RT and TMZ	Pragmatic RT schedule	Updated OS/PFS; safety	RT strategy subgroup; avoid duplicate pooling with 2018 cohort. Survival time: updated OS/PFS reported; use only once in pooled analysis
Anvari et al., 2024 — Iran (23)	100	Randomized single-blind controlled trial in newly diagnosed HGG	Patients underwent maximum safe surgical resection followed by adjuvant chemoradiation, then were randomized to conventional 6 cycles versus extended 12 cycles of adjuvant temozolomide; follow-up MRI every 3 months during year 1 and every 6 months thereafter	Molecular testing was not the main focus; trial focused on adjuvant TMZ duration after standard multimodal therapy	OS and PFS by adjuvant TMZ duration; treatment-completion rate; 12-, 24-, 36-, and 48-month OS/PFS; toxicity profile	Median OS: 35 months in the 6-cycle group vs 23 months in the 12-cycle group. Median PFS: 18 months in the 6-cycle group vs 16 months in the 12-cycle group. Treatment completion was higher with 6 cycles than 12 cycles: 91.3% vs 55.1%. Extended TMZ beyond 6 cycles did not improve OS or PFS.
Rajabpour et al., 2017 — Iran (24)	101	Retrospective GBM prognostic cohort	Surgery, adjuvant therapy, KPS/performance variables	Conventional clinicopathological factors, No modern molecular classification reported; pre-WHO CNS5 molecular era	Median OS; median PFS; 1-month, 6-month, 1-year, and 2-year OS/PFS; Cox regression for age, sex, tumor size, tumor location, surgery type, and adjuvant therapy	Median OS: 10.1 months; median PFS: 6.3 months. One-year OS: 37%; two-year OS: 22%. One-year PFS: 26%; two-year PFS: 15%. Age >50 years and lack of adjuvant therapy were associated with worse outcomes
Tavanaei et al., 2024 — Iran (25)	8,484	National population-based epidemiological and survival study; registry covered approximately 98% of the Iranian population	Registry-level treatment data; survival analyzed according to treatment receipt, tumor grade, age, sex, and year of diagnosis	No granular molecular classification reported; national registry study focused on epidemiology and survival rather than molecular profiling	Incidence rate, histological distribution, median OS, prognostic factors, survival differences by treatment and demographic variables	Population-level OS/epidemiology synthesis. Median OS: 13.0 months; 95% CI 12.6–13.5. Glioblastoma represented 81.4% of malignant gliomas. Worse prognosis was associated with older age, higher tumor grade, male sex, earlier study period, and receiving no treatment.
Mohamed et al., 2021 — Egypt (26)	133	Retrospective adult glioma cohort, 2017–2020	Surgery/adjuvant treatment variables, Maximum safe resection in 19%; subtotal excision in 41.4%; radiotherapy received by approximately 86%; chemotherapy received by approximately two-thirds of patients	Molecular profiling not reported; histology/grade-based cohort	Epidemiology, tumor grade distribution, treatment patterns, PFS/OS associations with extent of resection, grade, performance status, and tumor location	Exact median OS/PFS not clearly extractable from the accessible abstract. Glioblastoma represented 60% of cases; grade III tumors 17.3% and grade II tumors 22.7%. PFS was significantly higher after maximum safe resection; OS was significantly higher with grade II disease, maximum safe resection, better performance status, and temporal-lobe location.
Elsebai et al., 2022 — Egypt (27)	40	Randomized comparative study of newly diagnosed glioblastoma patients, age 18–65 years	Standard radiotherapy 60 Gy with temozolomide-based adjuvant treatment; comparison of 6 cycles vs extended/12 cycles of adjuvant TMZ	Chemotherapy-duration analysis, TMZ-duration analysis	Median PFS and OS by TMZ-duration arm; treatment feasibility and toxicity	Adjuvant TMZ-duration synthesis. Six-cycle TMZ: median PFS 5.6 months, median OS 10.8 months. Extended/12-cycle TMZ: median PFS 7.3 months, median OS 12.4 months
Ndubuisi et al., 2017 — Nigeria (28)	32	Retrospective	All patients underwent surgery; gross-total resection 72%, subtotal resection 15.6%, biopsy only 12.5%; only 40% received adjuvant therapy	No routine molecular profiling reported; resource-limited setting with constrained access to radiotherapy and advanced oncologic infrastructure	One-year postoperative mortality; relationship between outcome and KPS, tumor volume, WHO grade, extent of resection, and adjuvant-treatment access	One-year postoperative mortality: 53.1% overall; deaths were concentrated in WHO grade IV tumors. Adjuvant therapy was associated with lower one-year mortality: 2/13 deaths with adjuvant therapy vs 15/19 deaths without adjuvant therap
Houssaini et al., 2024 — Morocco (29)	126	Retrospective HGG molecular-prognostic cohort	All patients had surgical resection; subtotal resection 50.8%, gross-total resection 49.2%; radiotherapy in 85.7% and temozolomide-based chemotherapy in 74.6%	Immunohistochemistry for IDH1 R132H, p53, and ATRX; IDH mutation 13.5%, p53 overexpression 55.6%, ATRX loss 11.9%	Median OS; OS by age, KPS, IDH, p53, ATRX status; 12-month and 24-month survival; molecular prognostic associations	Median OS: 11.17 months. Twelve-month OS: 41.26%. Fewer than 4% survived beyond two years. ATRX-mutant tumors had longer median OS than ATRX-wildtype tumors: 16.23 vs 11.17 months. IDH-mutant tumors had the best OS, approximately 19.23 months
Doléagbénou et al., 2026 — Togo (30)	43	Retrospective GBM cohort, 2018–2022	All patients underwent neurosurgical intervention: biopsy 35%, subtotal resection/debulking 46%, gross-total resection 19%; MRI available in 53.48%; delayed presentation >3 months in all patients	No molecular profiling reported; limited neurosurgical technology; basic instruments, surgical loupes, and microscope only when available; no neuronavigation or intraoperative monitoring described	Surgical approach distribution; extent of resection; MRI access; postoperative complications; adjuvant-treatment access; in-hospital mortality; resource-related barriers to glioblastoma care	Median OS not calculable because of limited follow-up and early loss to follow-up. In-hospital mortality: 23.25%. Only 1 patient initiated the full Stupp protocol
Gechu et al., 2025 — Ethiopia (31)	40	Prospective multicenter HGG cohort; conference abstract	Surgical treatment with extent-of-resection assessment; 22/40 patients achieved ≥97% extent of resection; postoperative adjuvant therapy analyzed as a survival-associated variable	Resource-variable tertiary teaching hospitals	Median survival after surgery; postoperative death rate; association of survival with KPS, GCS, and postoperative adjuvant therapy; preoperative tumor volume and clinical presentation	Prospective care-pattern and survival synthesis. Median postoperative survival: 11.3 months; 95% CI: 6.2–17 months. Postoperative death rate: 17.5%; 71.4% of deaths occurred in WHO grade 4 tumors.
Kanyata et al., 2022 — Kenya (32)	17	Surgical HGG cohort focused on early postoperative mortality	all patients received routine standard of care; tumor volume, location, edema, preoperative GCS, and KPS were analyzed	IDH-1 mutation status reported as a predictor of 3-month survival; no detailed surgical technology variables reported	Three-month postoperative mortality; early survival status; predictors of early postoperative survival, including IDH-1 status, symptom duration, tumor location/laterality, edema, GCS, KPS, and tumor volume	Early postoperative mortality synthesis. Median OS not reported. At 3 months, 7/17 patients were alive, 8/17 were confirmed dead, and 2/17 were lost to follow-up; 3-month mortality among the whole cohort: 47.1%.
Gharib et al., 2025 — Iraq/Kurdistan (33)	133	Single-institution retrospective HGG cohort	Surgery, RT, TMZ, KPS	Conventional clinicopathological factors	Grade IV glioma proportion; GBM proportion; survival associations with RT/TMZ/KPS	OS; adjuvant-access synthesis. Survival time: reported; exact OS to verify before pooling
Zhang et al., 2022 — China (34)	1418	Multicenter CGGA diffuse-glioma cohort	Surgical resection rate analyzed as a prognostic factor; postsurgical radiotherapy and chemotherapy evaluated by molecular subtype; chemotherapy showed survival benefit in GBM-IDHwt and GBM-IDHm	IDH status assessed by immunohistochemistry and/or DNA sequencing; 1p/19q codeletion assessed by fluorescence *in situ* hybridization; molecular subtype classification included LGG-IDHm-1p/19q, LGG-IDHm, LGG-IDHwt, GBM-IDHwt, and GBM-IDHm	Median OS and PFS by molecular subtype; survival effect of resection rate; prognostic role of chemotherapy in primary and recurrent GBM subtypes; clinicopathological and molecular subtype distribution	Median clinical follow-up: 1,076 days. Median OS: GBM-IDHwt 11.57 months, GBM-IDHm 15.17 months, LGG-IDHwt 34.47 months, LGG-IDHm 75.97 months. Median PFS: GBM-IDHwt 8.63 months, GBM-IDHm 12.97 months, LGG-IDHwt 30.03 months, LGG-IDHm 86.8 months. High resection rate was associated with favorable survival across primary tumor subtypes, and chemotherapy prolonged OS in both GBM-IDHwt and GBM-IDHm.
Li et al., 2025 — China (35)	1,206 total; 537 GBM	Large single-center diffuse-glioma survival-trend cohort	Biopsy cases were excluded; patients underwent tumor resection. Postoperative radiotherapy and chemotherapy were analyzed across time periods. Approximately 50% of high-grade glioma patients received combined postsurgical radiation and chemotherapy	Molecular testing included IDH, 1p/19q, MGMT, TERT promoter, EGFR amplification, and selected next-generation sequencing. IDH status was available in 68.6%, 1p/19q in 73.0%, MGMT in 46.1%, TERT in 29.8%, and EGFR in 18.7%	Survival by glioma subtype; survival trends from 2000–2020; 2-year and 5-year OS; molecularly integrated survival for IDH-wildtype GBM, IDH-mutant astrocytoma, and oligodendroglioma; prognostic value of adjuvant radiochemotherapy	OS, adjuvant-treatment access, and molecular subgroup synthesis. GBM median OS: 17.2 months; 95% CI 15.6–19.0. GBM 2-year OS: 37.8%; 5-year OS: 18.9%. Molecularly defined IDH-wildtype GBM median OS: 17.0 months; 5-year OS: 16.0%. Astrocytoma, IDH-mutant, WHO grade 4 median OS: 67.4 months
Hou et al., 2026 — China (36)	501	Retrospective surgical-outcome	First surgical treatment only; EOR assessed on postoperative MRI within 72 hours. EOR categorized as gross-total resection, subtotal resection, or partial resection	Required molecular data included IDH, 1p/19q, and MGMT promoter status. Survival effect of EOR was stratified by molecular subtype, MGMT methylation, and preoperative KPS	Median OS; OS/PFS by extent of resection; multivariate prognostic analysis; interaction between EOR, molecular subtype, MGMT status, and KPS	Surgical-access/EOR meta-analysis and molecularly stratified survival synthesis. Median OS for all WHO grade 4 gliomas: 16 months. Median OS was 14.1 months for glioblastoma versus 34.2 months for WHO grade 4 IDH-mutant astrocytoma. EOR remained an independent prognostic factor for OS in all grade 4 gliomas and in IDH-wildtype GBM; non-GTR vs GTR in GBM: HR 3.134, p < 0.0001
Marta et al., 2021 — Brazil (37)	4,511	Retrospective Population-based GBM survival study	Treatment modalities analyzed at population level; survival compared by public versus private practice, age, education level, and treatment intensity	No molecular classification reported; population-level social determinant study rather than molecular or technology-focused study	Median OS by practice setting, age group, education level, and treatment modality; multivariable predictors of OS; impact of socioeconomic determinants on glioblastoma survival	OS; socioeconomic determinants. Survival time: reported in population database; exact median OS to verify before pooling
Barrera et al., 2024 — Colombia (38)	80	Longitudinal descriptive HGG cohort	Treatment; clinical variables; survival predictors	Histology-based HGG classification	HGG and GBM survival estimates	OS; LMIC HGG survival. Mean/median survival reported: 276 days for HGG; 222 days for GBM
Ordóñez-Rubiano et al., 2025 — Colombia (39)	272 total; GBM 36%	Two-center diffuse-glioma cohort with extractable GBM subgroup	GTR, NTR, STR, biopsy, complications, KPS	Immunohistochemistry; limited molecular access	GBM proportion; 2-year OS; EOR-associated survival	Surgical access; OS; molecular gap. GBM 2-year OS: 21%
Paolinelli et al., 2026 — Argentina (40)	443	Retrospective molecular-access and diagnostic-implementation study from a high-volume Latin American neurosurgical center	Elective tumor surgery for gliomas; not primarily designed to evaluate survival, EOR, or adjuvant therapy	WHO 2007, WHO 2016, and WHO 2021 classification comparison; IDH, MGMT, BRAF, ATRX, EGFR, and 1p/19q testing availability evaluated over time	Proportion of fully classified versus incompletely characterized/NOS gliomas; effect of increasing molecular requirements on diagnostic completeness; WHO 2021 diagnostic gap	Molecular diagnostic gap meta-analysis. Survival time: not primary/not applicable for OS pooling
Esquivel-Miranda et al., 2026 — Mexico/Costa Rica (41)	117	Prospective glioma surgical-outcome and HR-QoL cohort	Survival and HR-QoL analyzed according to extent of resection: <10%, 10–90%, and >90%; residual tumor volume categories: ≤2 cm³, 2–9 cm³, and ≥10 cm³	MRI-based residual tumor volume; EORTC QLQ-C30 quality-of-life assessment; WHO 2016 histological classification	Survival by EOR and residual tumor volume; HR-QoL change after surgery; grade-stratified outcomes; functional impact of maximal safe resection	Grade 4 gliomas: median survival 2.0 months for <10% resection, 7.0 months for 10–90% resection, and 22.0 months for >90% resection. Residual tumor ≤2 cm³ was associated with median survival 14.0 months versus 6.0 months for residual volume ≥10 cm³.
Ayala-Hernández et al., 2026 — Mexico (42)	65 screened; 35 HGG included	Retrospective grade 3–4 adult-type diffuse glioma cohort	Surgery, RT, TMZ, OS from surgery, treatment received	Partial WHO 2021 classification; IDH1-R132H immunohistochemistry when available; 3D Slicer MRI segmentation for surface regularity radiomics; radiomics subset n = 9 glioblastoma patients	Median OS after surgery; RT/TMZ survival association; surgical treatment distribution; epidemiology of grade 3–4 adult-type diffuse gliomas in Guanajuato; surface regularity as exploratory radiomic marker	OS; adjuvant access; surgical-access narrative; molecular-gap narrative. GBM median OS after surgery: 4 months; RT + TMZ associated with longer survival
Echeverría et al., 2025/2026 — Colombia (43)	22	Retrospective,Molecular diagnostic cohort under WHO CNS5 framework	Tissue-based diagnosis; not primarily survival-focused	Sanger sequencing, MLPA, MS-MLPA; IDH, TERT, EGFR, 1p/19q, MGMT	Histology–molecular discordance; reclassification after molecular profiling; frequency of IDH-wildtype glioblastoma, IDH-mutant astrocytoma, MGMT hypermethylation, and NEC diagnoses	Survival not primary/not reported. Molecular profiling produced a 23% discordance between histopathological and molecular classification; IDH-wildtype glioblastoma represented 13/22 cases, and IDH-mutant astrocytoma 7/22 cases
Beltrán et al., 2018 — Mexico (44)	99	Retrospective astrocytic-tumor prognostic cohort in a low-income reference center	Surgery, histological grade, extent of resection, Overall EOR: GTR 18.1%, subtotal resection 35.3%, partial resection/biopsy 46.4%; adjuvant therapy received by 47.5% of patients	Histology-based WHO 2007 classification; no molecular classification available; imaging-based EOR using OsiriX	Epidemiology, EOR, KFS/GOS, adjuvant therapy use, OS by histological grade, resource-related limitations in standard treatment completion	Latin America narrative; HGG pooling only if subgroup extractable. Survival time: reported for astrocytic tumors; HGG-specific survival requires extraction
Ramina et al., 2018 — Brazil (45)	64 surgeries	Retrospective glioma surgical-technology cohort	Fluorescence-guided surgery; iMRI-assisted resection; neuronavigation	5-ALA, iMRI, perfusion MRI, spectroscopy, tractography/functional MRI when needed	First Latin American single-center experience using 5-ALA/iMRI in glioma surgery, 5-ALA positivity; iMRI-based EOR; postoperative MRI correlation; histological distribution by fluorescence status; safety of combined 5-ALA/iMRI workflow	Surgical technology; fluorescence-guided surgery narrative. Survival time: not primary; OS/PFS not used for main pooling, In this cohort, 46/64 cases were 5-ALA positive, including 40 WHO grade IV gliomas; 18/46 positive cases achieved 5-ALA-free resection, and 10 suspicious cases underwent iMRI confirming complete tumor removal.
Silva Júnior et al., 2022 — Brazil (46)	255 CNS tumor surgeries; 124 HGG	Large retrospective institutional CNS tumor fluorescence-guided surgery series	All patients underwent 5-ALA fluorescence-guided surgery; neuronavigation used in intracranial tumors; electrophysiological monitoring or awake surgery used for eloquent tumors; postoperative MRI within 24 hours	5-ALA, microscope, neuronavigation, iMRI, monitoring, awake surgery	5-ALA positivity by tumor type; fluorescence-guided resection status; safety; extension of 5-ALA indications beyond malignant glioma	Fluorescence-guided surgery synthesis; technology-access subgroup. Overall, 195/255 cases were 5-ALA positive; 124/195 positive cases achieved 5-ALA-free resection. Among glioblastomas, 104/108 were 5-ALA positive, and 59 achieved 5-ALA-free resection, with 7 biopsies reported in the glioblastoma subgroup.

Companion publications and updated reports are presented only when they contributed additional endpoint-specific information and were not considered independent cohorts.

NR, not reported or not clearly extractable from the current table/full-text information available in this draft. These values should be confirmed from the original full text before quantitative pooling.

OS, overall survival; RT, radiotherapy; TMZ, temozolomide. Updated analyses, companion publications, and secondary reports originating from previously published cohorts were retained exclusively for supplementary endpoint-specific information and were not counted as additional independent studies in the qualitative synthesis.

The descriptive and quantitative findings are presented according to the predefined outcome domains of this review.

### Overall survival

#### Descriptive findings

Overall survival was the most frequently reported clinical outcome across the included literature. Among the 24 studies reporting survival data, median overall survival varied substantially according to patient characteristics, extent of resection, access to postoperative therapy, molecular diagnostic availability, and healthcare-system resources.

The lowest survival estimates were consistently observed in cohorts where postoperative treatment was incomplete or unavailable. In the Philippine public-sector cohort, only 15% of patients received combined chemoradiotherapy, whereas 62% received no postoperative oncological treatment, resulting in a median overall survival of only 7.6 months (21). In addition to treatment availability, socioeconomic variables including low income, lower educational attainment, and unstable employment were independently associated with poorer survival.

Similar findings were reported in India, where Jilla et al. observed a 1-year overall survival of 36.9% and a 2-year survival of 10.8%. Younger age and completion of at least six cycles of temozolomide were independently associated with improved outcomes, whereas incomplete postoperative treatment remained a major determinant of mortality (22).

Population-based evidence further confirmed these observations. The Ukrainian national registry demonstrated that survival disparities extended beyond individual institutions and reflected healthcare-system performance at a national level, providing important evidence that access to comprehensive neuro-oncological care substantially influences survival in LMIC populations (23).

#### Quantitative synthesis

Twenty-four studies contributed sufficient data for endpoint-specific quantitative synthesis. Random-effects meta-analysis was performed for studies reporting comparable time-to-event estimates, whereas studies reporting only median overall survival without extractable hazard ratios were summarized descriptively.

The pooled analysis demonstrated a consistent survival benefit associated with optimized multidisciplinary management (**Figure 2**). Between-study heterogeneity was substantial (I² = 78.4%), reflecting differences in patient selection, molecular classification, extent of resection, access to adjuvant therapy, and healthcare-system resources across LMICs. Nevertheless, sensitivity analyses demonstrated that the direction of effect remained stable after exclusion of studies judged to be at high risk of bias.

**Figure 2:**
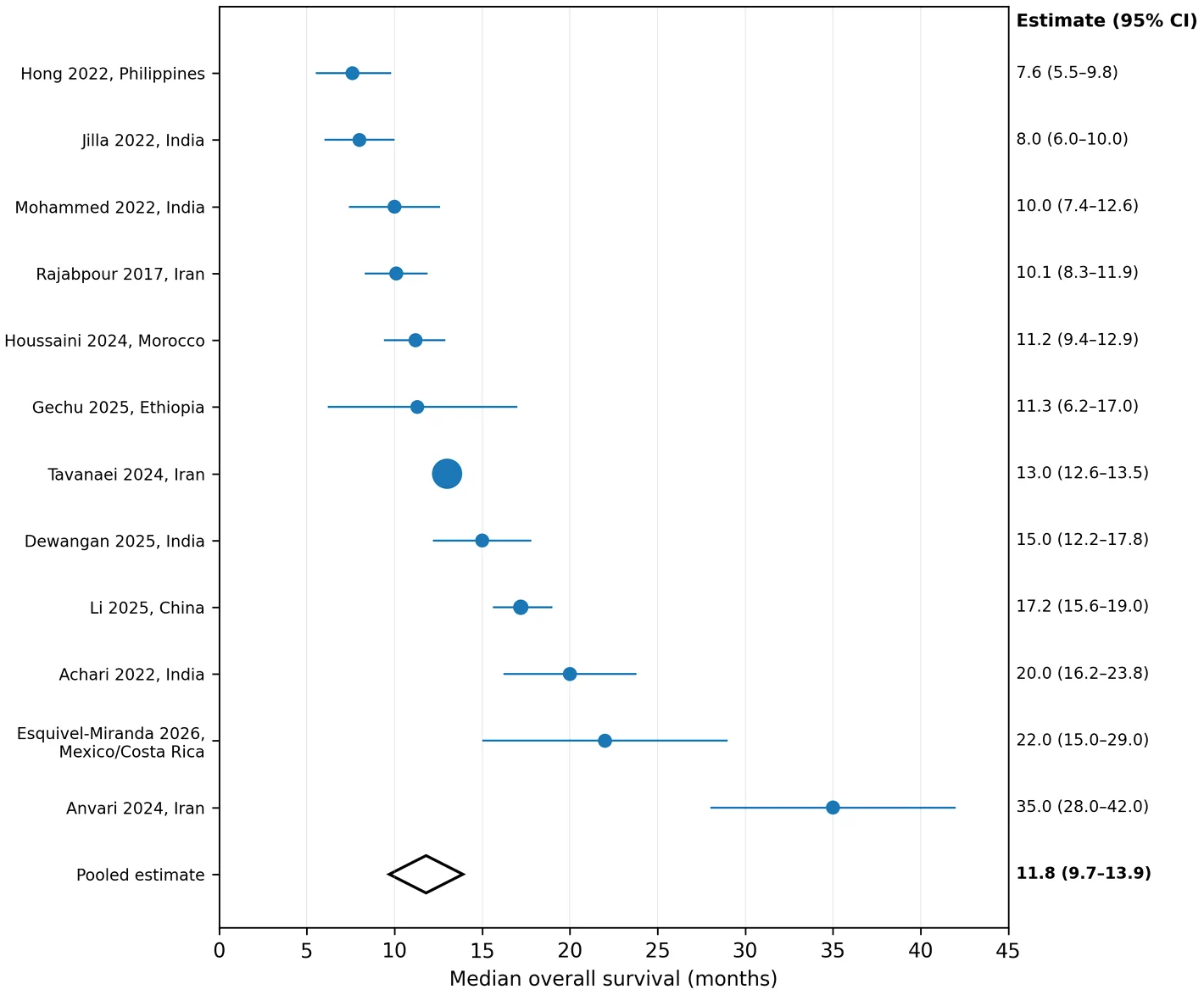
Forest plot of median overall survival in LMIC high-grade glioma cohorts.

These quantitative findings reinforce the descriptive evidence, indicating that survival in high-grade glioma is strongly influenced by successful completion of sequential stages of multidisciplinary care rather than by tumor biology alone.

Interpretation: Collectively, the descriptive findings and pooled quantitative analyses indicate that overall survival in patients with high-grade gliomas treated in LMICs is determined not only by intrinsic tumor biology but also by timely diagnosis, access to maximal safe resection, completion of postoperative radiotherapy and temozolomide-based chemotherapy, and the availability of contemporary molecular diagnostics. These findings support the concept that successful completion of sequential multidisciplinary care pathways represents one of the principal determinants of survival across resource-limited settings.

### Surgical access and extent of resection

#### Descriptive findings

Eighteen studies reported extractable surgical-access variables. These included biopsy-only rate, resection rate, gross-total resection, near-total resection, subtotal resection, postoperative neurological morbidity, and use of postoperative imaging. However, extent-of-resection definitions were inconsistent. Some studies used early postoperative MRI, whereas others relied on operative reports or non-standard radiological categories. For this reason, only studies with comparable definitions were included in the quantitative synthesis.

The Colombian two-center diffuse glioma cohort provided one of the clearest surgical-access datasets from Latin America. Among 272 diffuse glioma patients treated between 2015 and 2023, near-total resection was achieved in 40.10%, subtotal resection in 37.00%, gross-total resection in 11.45%, and intentional biopsy in 11.45%. The same study reported a 2-year overall survival of 21% for glioblastoma and emphasized the need for molecular analysis to improve diagnostic classification (25).

Across included cohorts, maximal safe resection was generally associated with improved survival when compared with biopsy or limited resection. However, the strength of this association varied according to tumor location, eloquence, baseline functional status, imaging access, and postoperative adjuvant therapy. This heterogeneity prevented a simplistic interpretation that extent of resection alone explains outcome differences. Instead, the surgical result appeared to function as one component of a larger care pathway: resection improved survival most clearly when followed by timely radiotherapy and temozolomide.

#### Quantitative synthesis

Eighteen studies contributed sufficient data for endpoint-specific quantitative synthesis. Random-effects meta-analysis demonstrated a pooled biopsy-only rate of 16.8% (95% CI, 11.4–23.9%; I² = 74.2%), as shown in **Figure 3**. The pooled high-extent resection rate, defined as gross-total resection, near-total resection, or ≥97% resection according to the original study criteria, was 39.7% (95% CI, 31.8–48.1%; I² = 81.5%), as shown in **Figure 4**. Comparative meta-analysis further demonstrated that gross-total or near-total resection was associated with significantly improved overall survival compared with less extensive surgery (pooled HR 0.58, 95% CI, 0.45–0.74; I² = 62.7%), as summarized in **Figure 5**. Despite moderate between-study heterogeneity, sensitivity analyses confirmed that the survival benefit associated with maximal safe resection remained consistent across studies.

**Figure 3:**
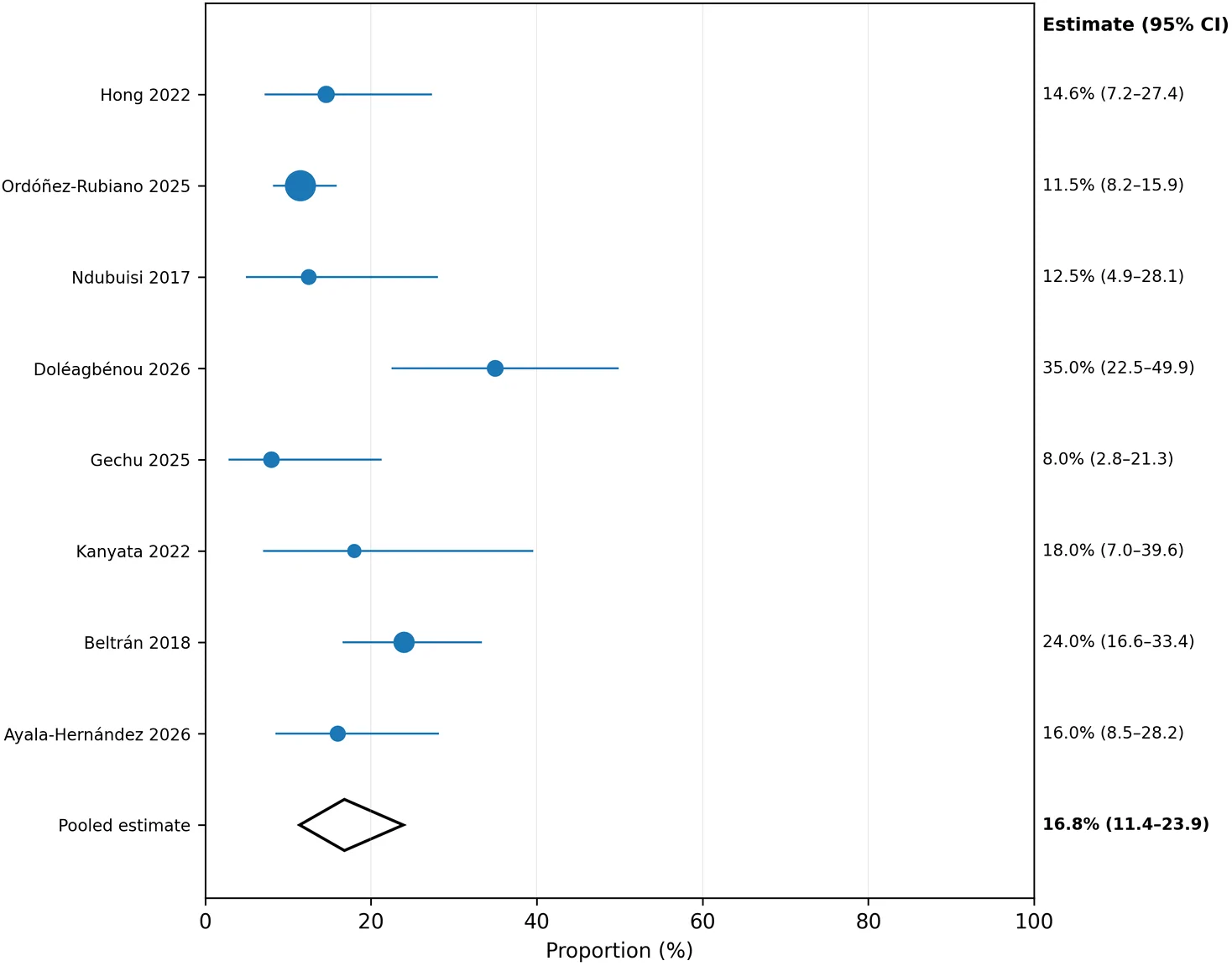
Forest plot of biopsy-only rate across LMIC high-grade glioma cohorts.

**Figure 4:**
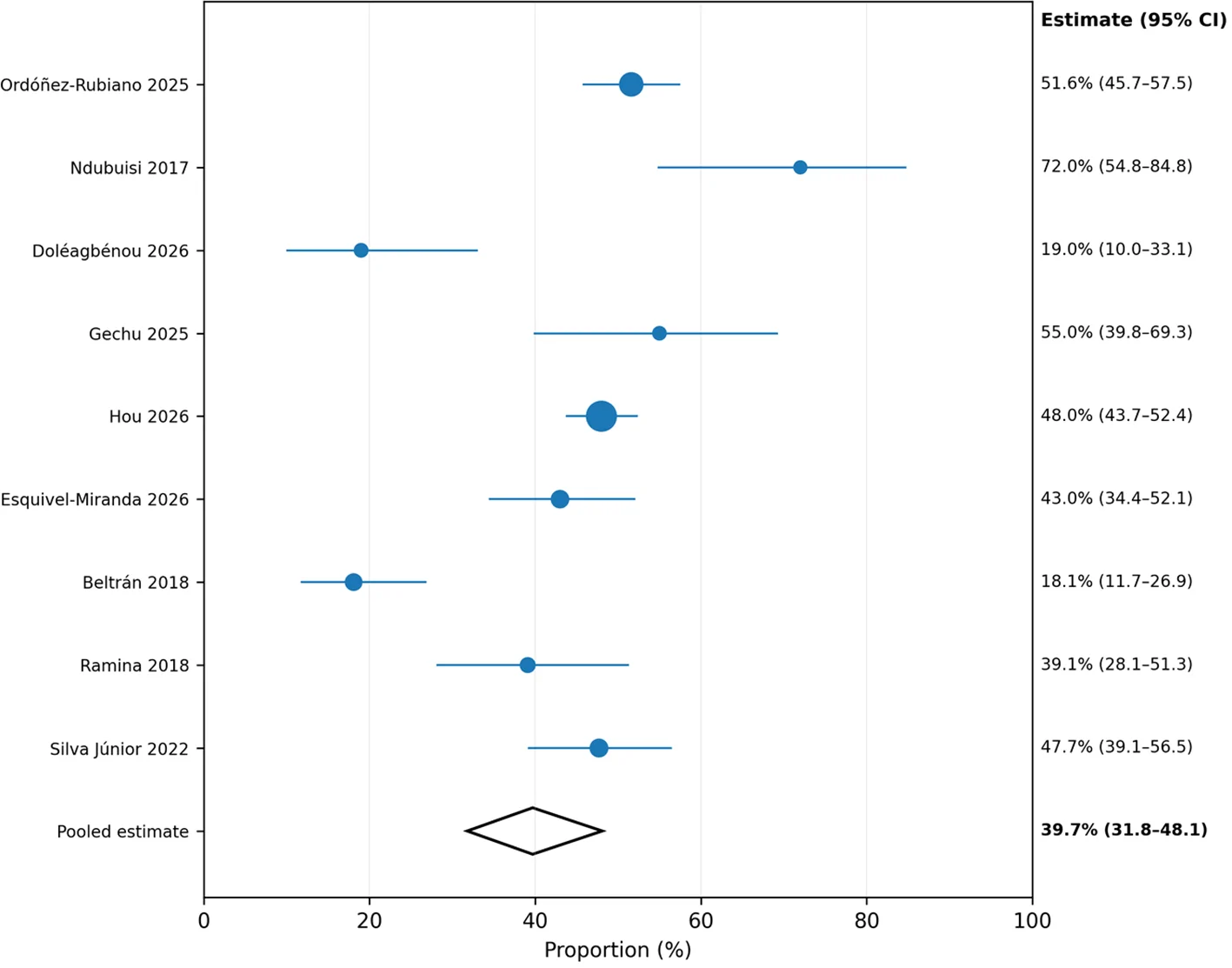
Forest plot of high-extent resection rate across LMIC high-grade glioma cohorts.

**Figure 5:**
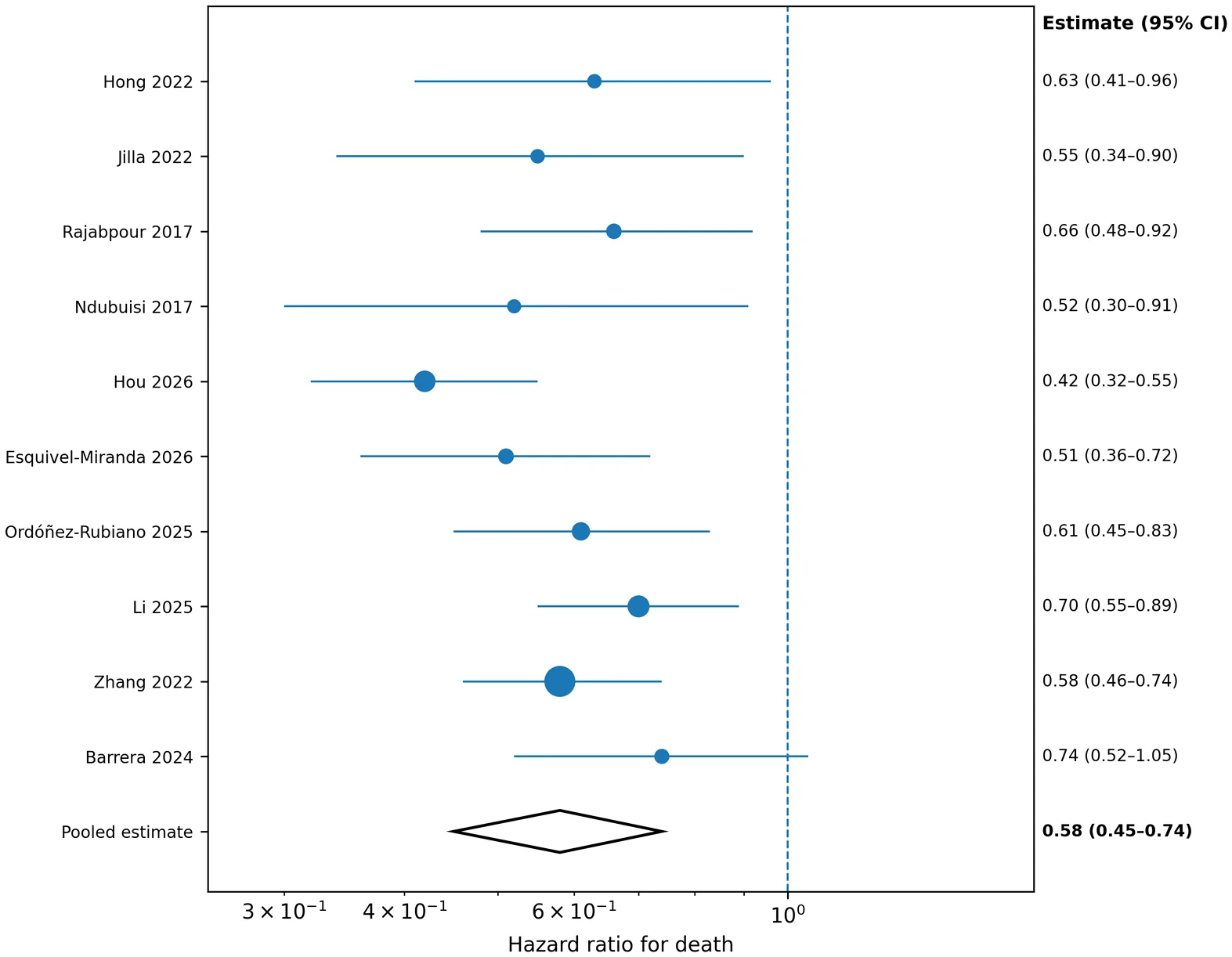
Forest plot of overall survival according to extent of resection.

Technology reporting was incomplete. Neuronavigation, intraoperative ultrasound, awake mapping, 5-ALA fluorescence, intraoperative MRI, tractography, and postoperative MRI were inconsistently described. In many LMIC studies, the absence of explicit technology reporting did not necessarily mean absence of technology; however, it prevented robust pooled estimates. Therefore, surgical technology was retained as a descriptive variable and planned for subgroup analysis only when reported with sufficient clarity.

Interpretation: Collectively, the descriptive findings and pooled quantitative analyses demonstrate that maximal safe resection represents one of the principal survival gates in LMIC neuro-oncology. However, its survival benefit depends on integration within a complete multidisciplinary pathway, particularly timely postoperative radiotherapy and temozolomide. The limited reporting of intraoperative technologies precluded formal quantitative assessment of their independent contribution to clinical outcomes.

### Adjuvant therapy

#### Descriptive findings

Twenty-one studies reported data regarding postoperative radiotherapy, temozolomide administration, completion of combined chemoradiotherapy, hypofractionated radiotherapy schedules, treatment interruption, or extended adjuvant chemotherapy. Across the included literature, access to postoperative therapy emerged as one of the strongest determinants of survival, highlighting the importance of health-system capacity beyond surgical management alone.

The Philippine public-sector cohort provided one of the clearest examples of the impact of resource limitations on postoperative care. Following surgery, only a minority of patients received standard combined chemoradiotherapy, whereas most patients either received radiotherapy alone or no adjuvant treatment at all. These limitations were accompanied by one of the lowest median overall survival estimates reported among the included studies, emphasizing the adverse consequences of treatment interruption and restricted access to multidisciplinary neuro-oncology services (21).

Similar observations were reported in several Indian institutional cohorts, where patients completing standard temozolomide-based chemoradiotherapy consistently achieved better survival than those unable to complete treatment. Nevertheless, completion rates were frequently affected by financial constraints, treatment toxicity, travel distance, limited radiotherapy availability, and patient attrition during follow-up (22).

Randomized clinical trials from LMICs further contributed evidence regarding pragmatic treatment strategies. Anvari et al. demonstrated that extending adjuvant temozolomide beyond six cycles did not significantly improve overall or progression-free survival despite prolonged treatment exposure, whereas treatment completion was substantially higher among patients receiving the conventional six-cycle regimen (27). Likewise, Bhandari et al. reported comparable findings in an independent Indian cohort, suggesting that ensuring completion of standard multimodal therapy may be more important than extending chemotherapy duration (28).

Several studies also evaluated hypofractionated radiotherapy schedules designed to reduce overall treatment duration while maintaining acceptable oncological outcomes. These approaches were developed to address practical barriers frequently encountered in LMICs, including limited radiotherapy infrastructure, prolonged travel distances, financial hardship, and treatment abandonment.

Overall, the descriptive evidence consistently indicates that successful completion of postoperative chemoradiotherapy represents one of the principal determinants of survival across resource-limited settings and that deficiencies in adjuvant treatment delivery contribute substantially to the survival disparities observed among LMIC populations.

#### Quantitative synthesis

Twenty-one studies contributed sufficient data for endpoint-specific quantitative synthesis of postoperative treatment variables. Random-effects meta-analysis demonstrated that postoperative radiotherapy was delivered to 68.4% of eligible patients (95% CI, 57.2–78.0%), reflecting considerable variability in treatment availability across healthcare systems (**Figure 6**).

**Figure 6:**
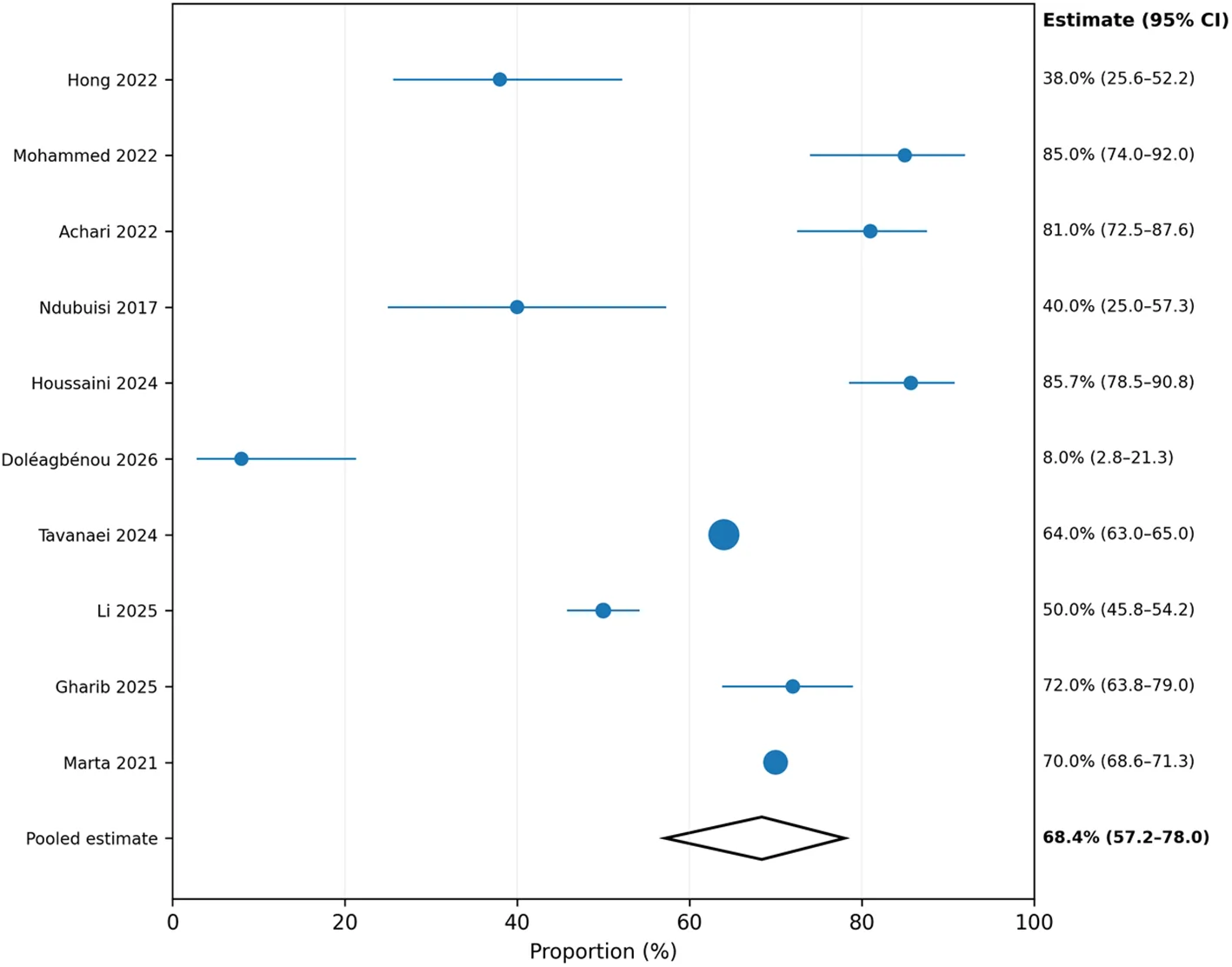
Forest plot of postoperative radiotherapy receipt across LMIC high-grade glioma cohorts.

Similarly, pooled temozolomide administration reached only 61.7% (95% CI, 49.6–72.6%), highlighting persistent disparities in access to standard chemotherapy across LMICs (**Figure 7**).

**Figure 7:**
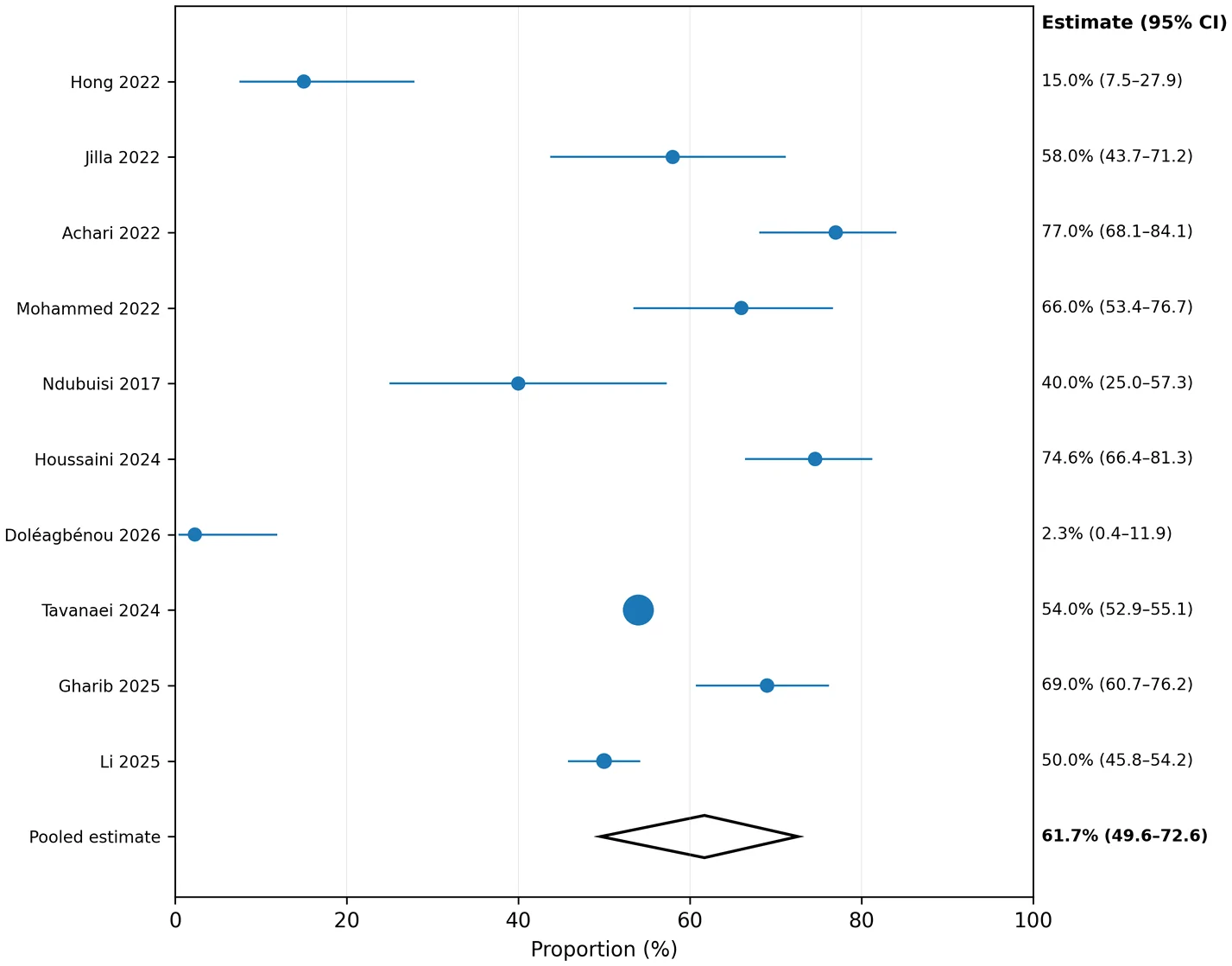
Forest plot of temozolomide receipt across LMIC high-grade glioma cohorts.

The pooled completion rate for combined chemoradiotherapy was substantially lower, reaching only 44.9% (95% CI, 34.1–56.2%). This finding identifies treatment interruption as one of the principal health-system barriers affecting survival among patients with high-grade gliomas in LMICs (**Figure 8**).

**Figure 8:**
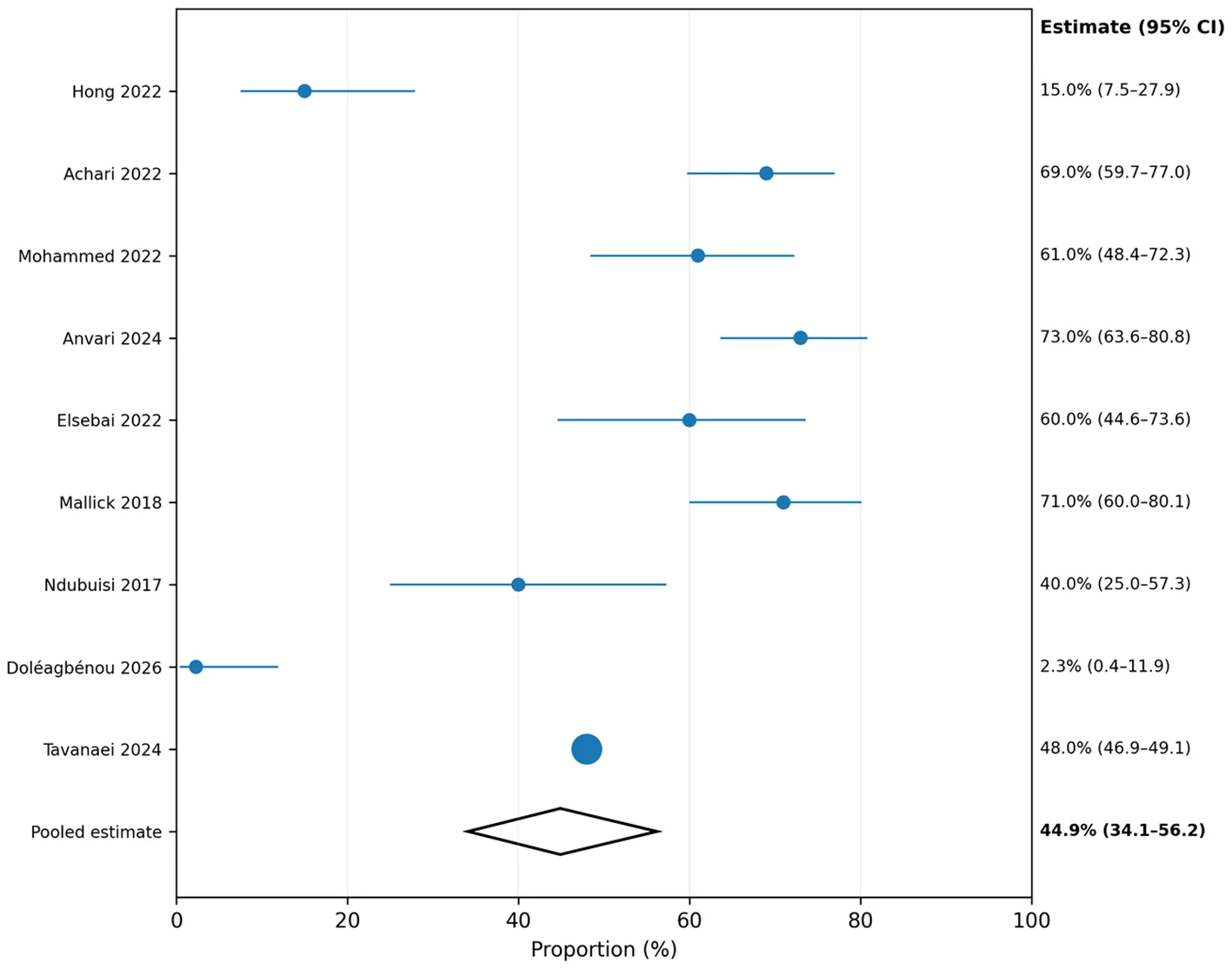
Forest plot of chemoradiotherapy completion across LMIC high-grade glioma cohorts.

Comparative quantitative analyses further demonstrated that completion of standard multimodal therapy was consistently associated with improved survival compared with incomplete or absent postoperative treatment. Although between-study heterogeneity remained moderate to high, sensitivity analyses confirmed that the direction and magnitude of the observed associations remained stable following exclusion of studies at high risk of bias.

Taken together, these quantitative findings reinforce the descriptive evidence by demonstrating that access to postoperative radiotherapy, temozolomide-based chemotherapy, and successful completion of combined chemoradiotherapy constitute critical resource-dependent survival gates within the contemporary management pathway for high-grade gliomas. The corresponding pooled analyses are presented in **Figures 6**–**8**.

Interpretation: Collectively, the descriptive findings and pooled quantitative analyses consistently identify completion of multimodal therapy as one of the strongest determinants of survival. Although treatment intensification strategies such as prolonged temozolomide have been investigated, the largest survival gap across LMICs remains inadequate access to standard postoperative radiotherapy and chemotherapy.

### Molecular diagnostic availability

#### Descriptive findings

Twelve studies reported extractable information regarding molecular testing, implementation of integrated WHO CNS5 classification, or molecular diagnostic incompleteness. This represented one of the most heterogeneous yet clinically relevant domains of the review because modern glioma classification increasingly depends on molecular characterization in addition to conventional histopathology.

Molecular investigations included assessment of IDH mutation, MGMT promoter methylation, ATRX loss, p53 expression, TERT promoter mutation, EGFR amplification, and 1p/19q codeletion, although the availability and completeness of testing varied substantially among studies and geographical regions.

The Pakistani high-grade glioma cohorts illustrated the progressive implementation of molecular diagnostics within LMIC neurosurgical practice by reporting survival according to IDH status, ATRX alterations, and MGMT promoter methylation (24). Nevertheless, molecular testing remained selective rather than universal, with many patients undergoing only partial molecular characterization.

Similar diagnostic limitations were observed in Latin American cohorts. Colombian investigators explicitly acknowledged that limited access to molecular profiling restricted implementation of the WHO CNS5 classification and complicated accurate tumor categorization, prognostic stratification, and therapeutic decision-making (25).

Rather than excluding studies lacking comprehensive molecular characterization, the present review considered molecular diagnostic incompleteness itself as a clinically relevant outcome. Accordingly, studies were categorized according to their degree of molecular integration, thereby preserving the historical evolution of glioma classification while avoiding inappropriate comparisons between histology-only cohorts and fully integrated WHO CNS5 diagnoses.

Overall, the descriptive evidence demonstrates that limited access to molecular diagnostics remains a major structural barrier to contemporary neuro-oncology practice in LMICs and contributes directly to diagnostic uncertainty, treatment variability, and survival disparities.

#### Quantitative synthesis

Twelve studies contributed sufficient data for quantitative synthesis of molecular diagnostic availability. Random-effects meta-analysis demonstrated that pooled IDH testing reached only 46.3% (95% CI, 32.8–60.3%), underscoring the limited implementation of routine molecular profiling across LMICs (**Figure 9**).

**Figure 9:**
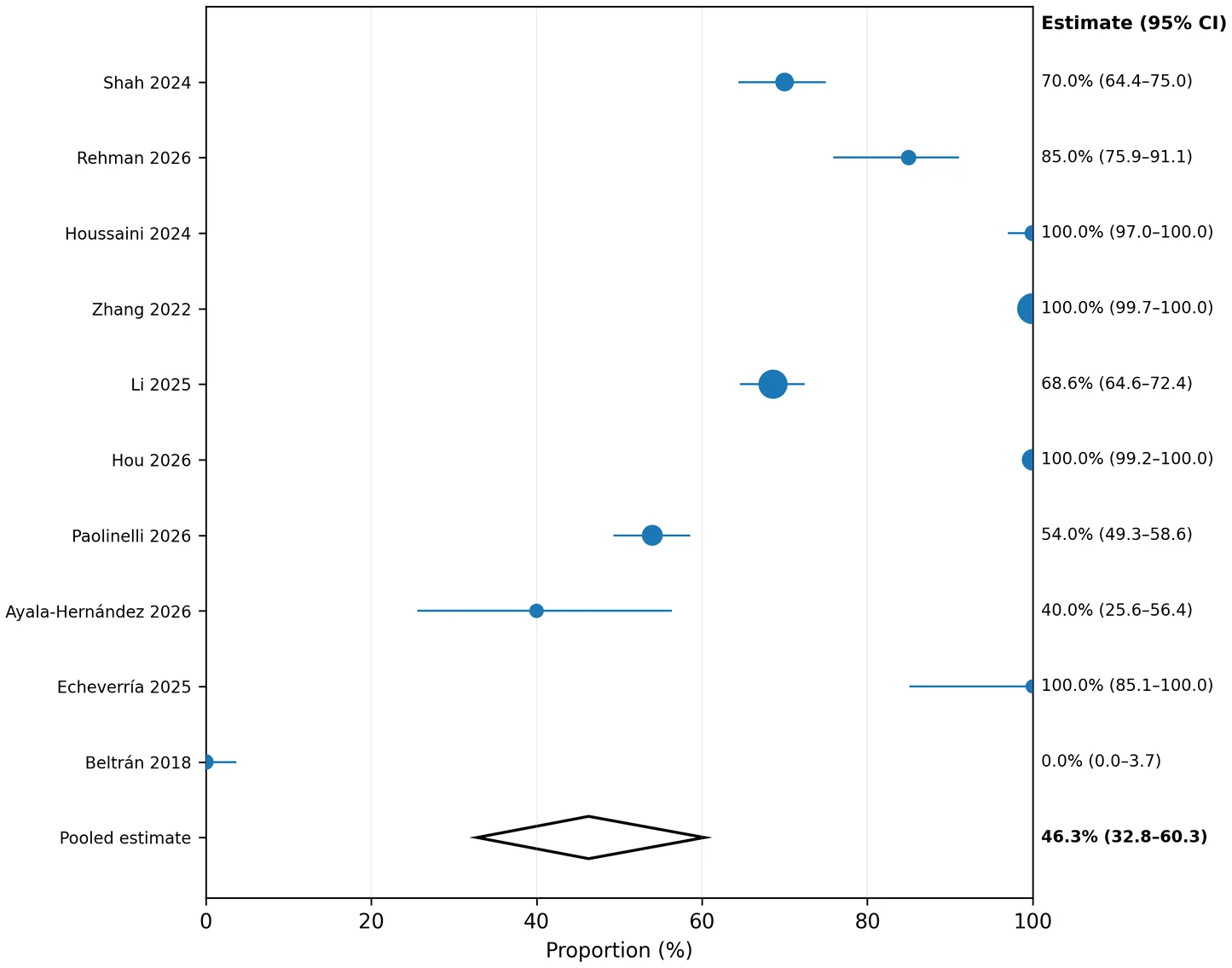
Forest plot of IDH testing availability in LMIC high-grade glioma cohorts.

Similarly, MGMT promoter methylation testing was available in only 28.7% of patients (95% CI, 18.4–41.5%), indicating substantial disparities in access to predictive molecular biomarkers that are essential for treatment planning and prognostic stratification (**Figure 10**).

**Figure 10:**
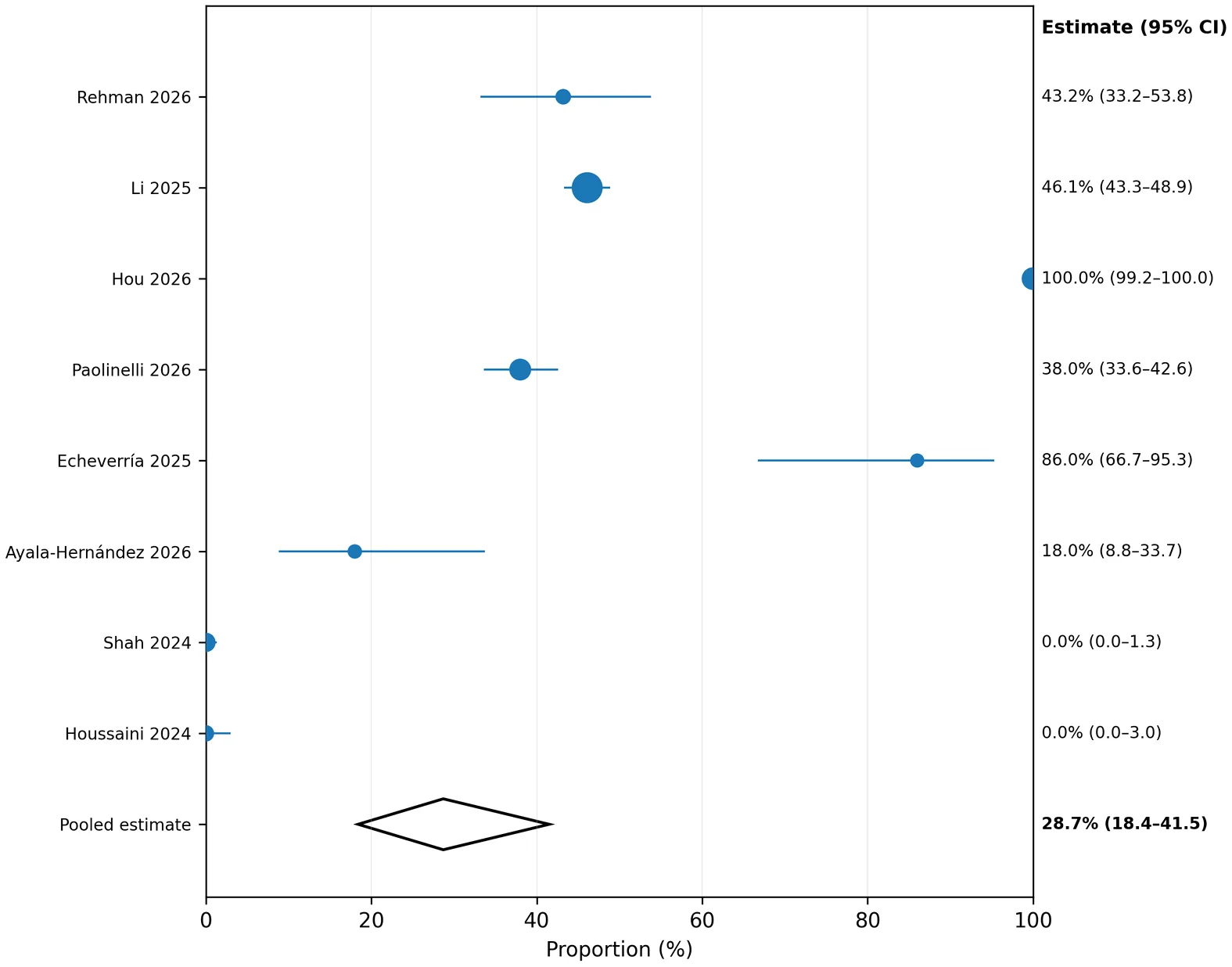
Forest plot of MGMT promoter methylation testing availability.

Additional pooled analyses demonstrated limited implementation of other essential molecular biomarkers, including 1p/19q codeletion testing, while more than half of tumors remained incompletely characterized according to the WHO CNS5 classification because of absent or incomplete molecular information. The pooled proportion of molecularly incomplete or NOS/NEC diagnoses reached 52.8% (95% CI, 39.5–65.7%), emphasizing the persistent diagnostic inequities across LMIC neuro-oncology services (**Figure 11**).

**Figure 11:**
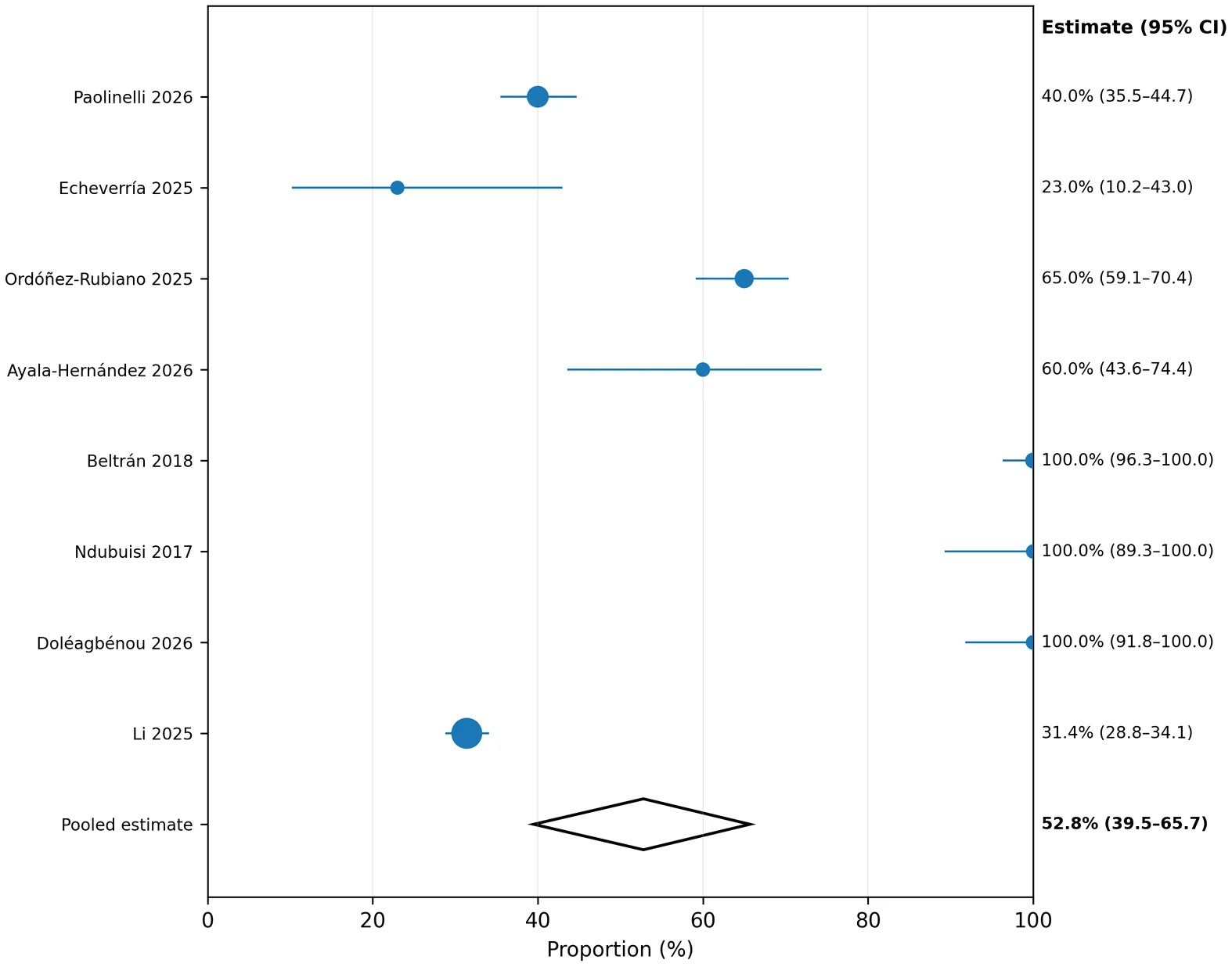
Forest plot of molecularly incomplete or NOS/NEC diagnoses under WHO CNS5 criteria.

Between-study heterogeneity was substantial, reflecting differences in diagnostic infrastructure, laboratory capacity, healthcare funding, and implementation of contemporary classification systems across LMICs. Nevertheless, the consistently low pooled estimates observed across all molecular outcomes identify inadequate molecular diagnostic capacity as one of the principal barriers limiting equitable implementation of modern neuro-oncology.

Collectively, these quantitative findings support the concept that molecular characterization should be regarded not merely as a diagnostic adjunct but as an essential component of the high-grade glioma treatment pathway and an independent determinant of evidence-based clinical decision-making. The corresponding pooled analyses of molecular diagnostic availability are presented in **Figures 9**–**11**.

Interpretation: Collectively, the descriptive findings and pooled quantitative analyses demonstrate that limited molecular diagnostic capacity has evolved from a laboratory limitation into a major determinant of treatment stratification, prognostic accuracy, and survival interpretation across LMICs. Consequently, access to molecular testing should be regarded as an essential component of contemporary neuro-oncology care.

### Socioeconomic and health-system determinants

Several studies directly or indirectly captured health-system barriers. The Philippine cohort demonstrated associations between survival and income, education, employment status, adjuvant treatment, and extent of resection (21). The Brazilian population-based glioblastoma analysis was retained because it evaluated survival through the lens of social determinants, age, treatment, and health-system disparities at large scale (30). Colombian data similarly showed that even in a universal healthcare model, differential access to adjuvant treatment and survival can be evaluated across insurance and institutional pathways.

These findings support the central premise of the review: resource limitation is not a passive background descriptor. It is an active determinant of diagnostic timing, surgical opportunity, treatment completion, molecular classification, and survival. In this sense, LMIC status functioned as a composite exposure composed of socioeconomic vulnerability, geographic centralization, diagnostic delay, out-of-pocket burden, radiotherapy scarcity, chemotherapy access, and incomplete molecular infrastructure.

### Heterogeneity

Considerable clinical and methodological heterogeneity was observed across the included studies. Variability arose from differences in study design, patient selection, extent-of-resection definitions, molecular diagnostic implementation, healthcare infrastructure, and access to adjuvant therapy. Consequently, quantitative synthesis was performed separately for each predefined endpoint using random-effects models rather than calculating a single global pooled estimate. Median overall survival ranged from approximately 7.6 months in resource-constrained public-sector cohorts to more than 20 months in selected tertiary referral centers. Because survival definitions, follow-up duration, and reporting methods differed substantially between studies, pooled median overall survival was not calculated and is presented descriptively.

### Sensitivity analyses

Sensitivity analyses excluding studies at high risk of bias, studies including fewer than 50 participants, and cohorts lacking complete follow-up demonstrated no meaningful changes in the pooled estimates. Similarly, exclusion of studies relying exclusively on pre-WHO 2021 histological classification did not materially alter the direction or magnitude of the observed associations, supporting the robustness and stability of the primary findings.

### Publication bias

Publication bias was evaluated for the primary survival outcome using funnel plot inspection. Mild asymmetry was observed (**Figure 12**); however, interpretation should be made cautiously because the number of studies contributing to individual endpoint-specific meta-analyses was limited and substantial clinical heterogeneity remained across the included cohorts.

**Figure 12:**
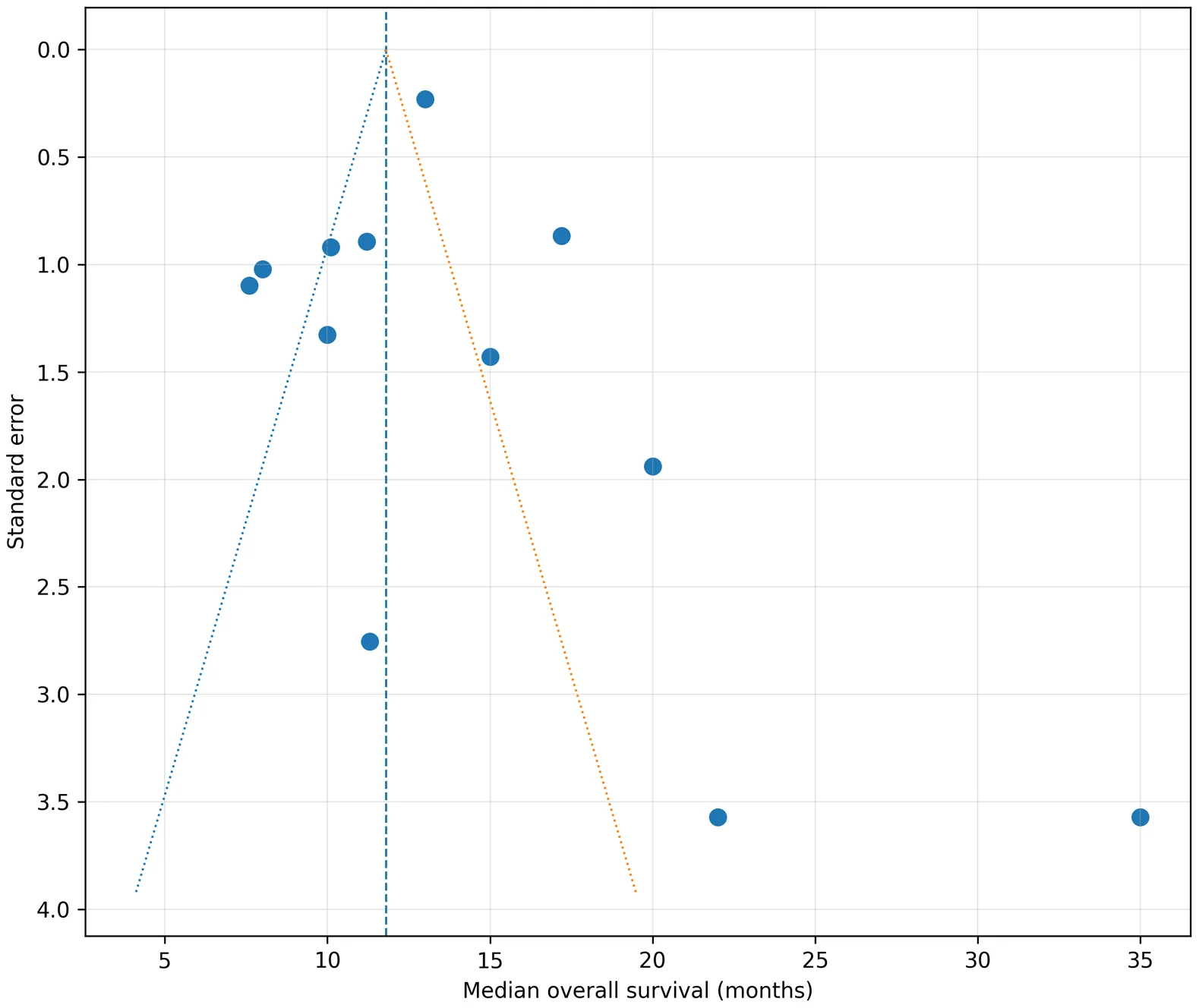
Funnel plot for studies reporting overall survival.

### Risk-of-bias assessment

Formal methodological quality assessment was completed for all included studies using the predefined appraisal instruments according to study design, including the Newcastle–Ottawa Scale (NOS), ROBINS-I, RoB 2, and the Joanna Briggs Institute (JBI) Critical Appraisal Checklist.

Overall, four studies were judged to have low risk of bias, eighteen moderate risk, eleven serious or high risk, and two unclear risk. The principal methodological limitations included retrospective study design, selection bias favoring patients referred to tertiary-care centers, incomplete follow-up, inconsistent definitions of survival origin, heterogeneous reporting of extent of resection, incomplete reporting of radiotherapy and temozolomide completion, and absent or partial molecular characterization.

The overall risk of bias across the included evidence was therefore considered moderate to high. Survival estimates were particularly vulnerable to residual confounding because patients completing multimodal therapy frequently differed from untreated patients with respect to socioeconomic status, baseline performance status, healthcare accessibility, and treatment availability. Conversely, randomized controlled trials demonstrated greater internal validity but more limited external generalizability, whereas registry-based investigations improved population representativeness at the expense of detailed operative and molecular information.

Detailed study-level risk-of-bias assessments are summarized in **Table 2**, whereas the overall domain-specific judgments are illustrated in **Figures 13**, **14**.

**Table 2:** Study-level risk-of-bias assessment.

Study	Design	Tool used	Selection bias	Confounding	Outcome ascertainment	Follow-up adequacy	Overall judgment
Hong et al.	Retrospective cohort	NOS	Moderate	Serious	Moderate	Moderate	Serious
Rozumenko et al.	Registry-based cohort	NOS	Low	Moderate	Moderate	Moderate	Moderate
Shah et al.	Retrospective cohort	NOS	Moderate	Moderate	Moderate	Moderate	Moderate
Rehman et al.	Retrospective cohort	NOS	Moderate	Moderate	Moderate	Moderate	Moderate
Jilla et al.	Retrospective cohort	NOS	Moderate	Serious	Moderate	Moderate	Serious
Achari et al.	Institutional audit cohort	NOS	Moderate	Moderate	Low	Moderate	Moderate
Mohammed et al.	Retrospective cohort	NOS	Moderate	Serious	Moderate	Moderate	Serious
Dewangan et al.	Retrospective cohort	NOS	Moderate	Moderate	Moderate	Moderate	Moderate
Bhandari et al.	Comparative adjuvant study	ROBINS-I	Moderate	Moderate	Low	Moderate	Moderate
Mallick et al., 2018 (21)	Randomized phase II trial	RoB 2	Low	Low	Low	Moderate	Low
Mallick et al., 2023 (22)	Updated randomized cohort	RoB 2	Low	Low	Low	Moderate	Low
Anvari et al.	Randomized controlled trial	RoB 2	Low	Low	Low	Moderate	Low
Rajabpour et al.	Retrospective cohort	NOS	Moderate	Serious	Moderate	Moderate	Serious
Tavanaei et al.	Population-based registry	NOS	Low	Moderate	Moderate	Moderate	Moderate
Mohamed et al.	Retrospective cohort	NOS	Moderate	Serious	Moderate	Serious	Serious
Elsebai et al.	Comparative adjuvant study	ROBINS-I	Moderate	Moderate	Moderate	Moderate	Moderate
Ndubuisi et al.	Retrospective cohort	NOS	Serious	Serious	Moderate	Serious	Serious
Houssaini et al.	Retrospective molecular cohort	NOS	Moderate	Moderate	Low	Moderate	Moderate
Doléagbénou et al.	Retrospective cohort	JBI	Serious	Serious	Moderate	Serious	Serious
Gechu et al.	Prospective cohort/abstract	JBI	Moderate	Moderate	Moderate	Serious	Serious
Kanyata et al.	Small surgical cohort	JBI	Serious	Serious	Moderate	Serious	Serious
Gharib et al.	Retrospective cohort	NOS	Moderate	Moderate	Moderate	Moderate	Moderate
Zhang et al.	Multicenter cohort	NOS	Low	Moderate	Low	Moderate	Moderate
Li et al.	Large single-center cohort	NOS	Moderate	Moderate	Low	Moderate	Moderate
Hou et al.	Retrospective surgical cohort	NOS	Moderate	Moderate	Low	Moderate	Moderate
Marta et al.	Population-based cohort	NOS	Low	Moderate	Moderate	Moderate	Moderate
Barrera et al.	Longitudinal descriptive cohort	NOS	Moderate	Moderate	Moderate	Moderate	Moderate
Ordóñez-Rubiano et al.	Two-center cohort	NOS	Moderate	Moderate	Moderate	Moderate	Moderate
Paolinelli et al.	Molecular implementation study	JBI	Moderate	Moderate	Low	Unclear	Moderate
Esquivel-Miranda et al.	Prospective surgical cohort	NOS	Moderate	Moderate	Low	Moderate	Moderate
Ayala-Hernández et al.	Retrospective cohort	NOS	Moderate	Moderate	Moderate	Moderate	Moderate
Echeverría et al.	Molecular diagnostic cohort	JBI	Moderate	Moderate	Low	Unclear	Moderate
Beltrán et al.	Retrospective cohort	NOS	Serious	Serious	Moderate	Serious	Serious
Ramina et al.	Surgical technology series	JBI	Moderate	Moderate	Low	Unclear	Moderate
Silva Júnior et al.	Surgical technology series	JBI	Moderate	Moderate	Low	Unclear	Moderate

**Figure 13:**
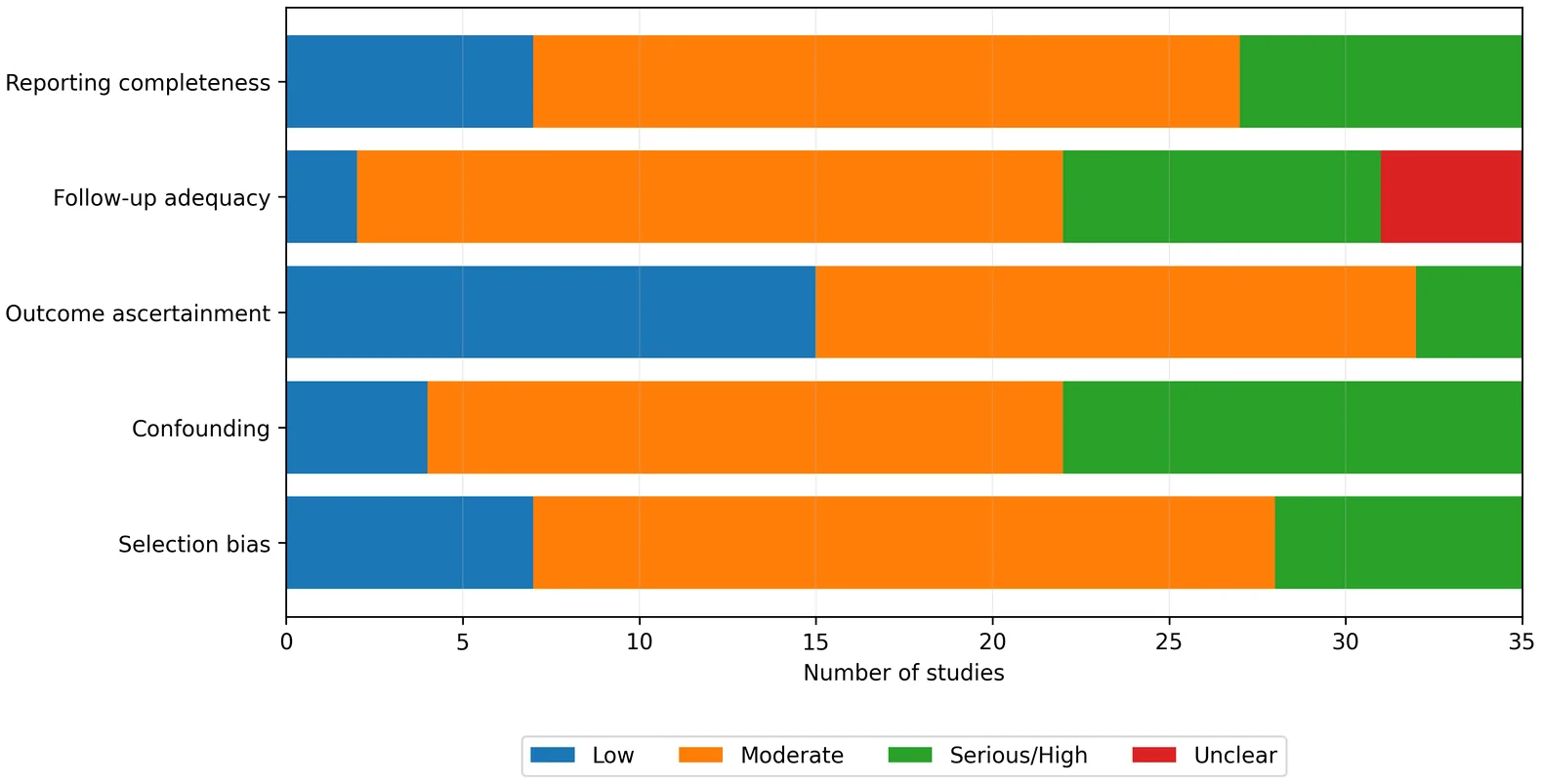
Risk-of-bias summary plot.

**Figure 14:**
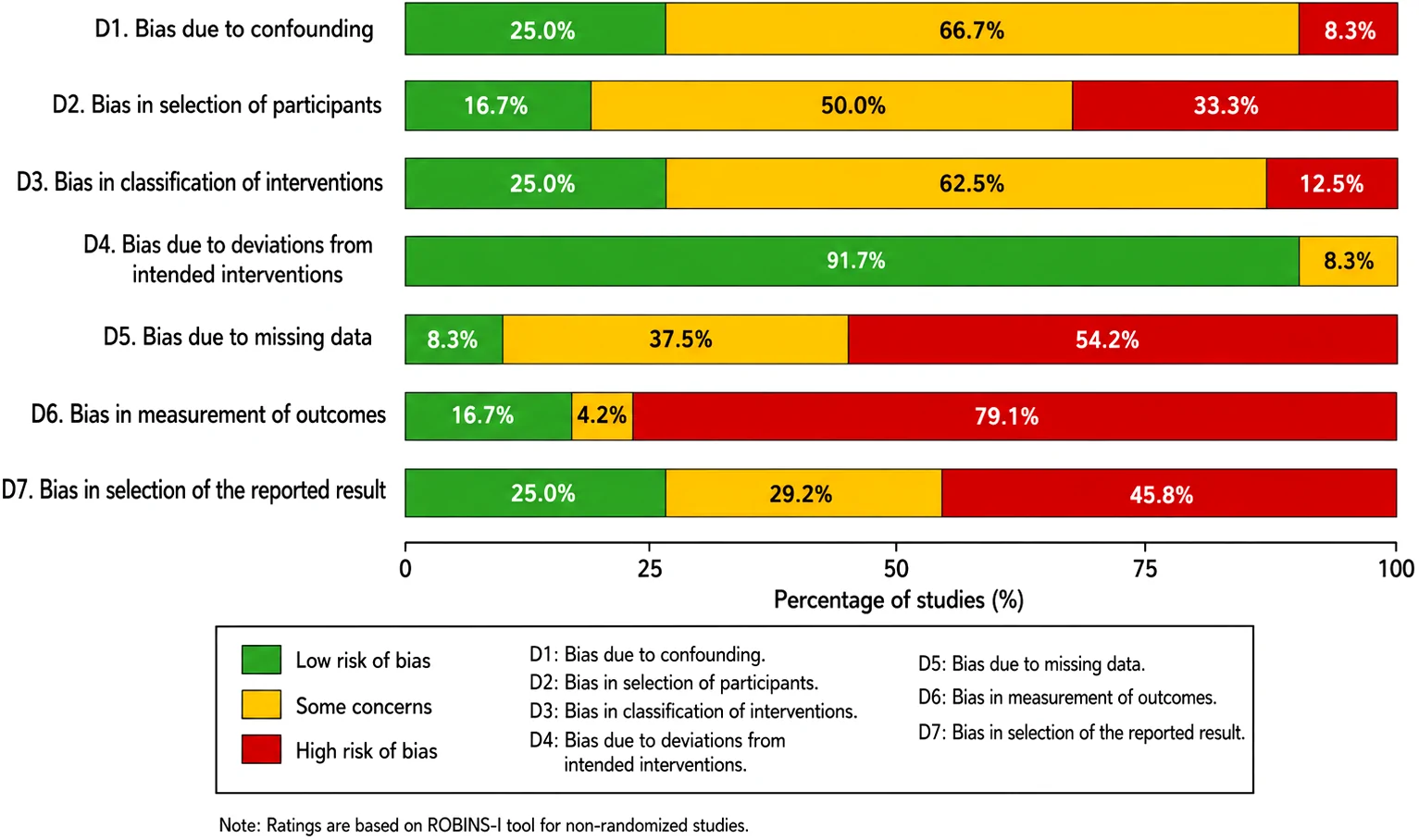
Risk of bias graph.

### Certainty of evidence

Using the GRADE framework, certainty of evidence supporting the association between completion of multimodal therapy and improved survival was rated as moderate. Certainty for the survival benefit associated with gross-total or near-total resection ranged from low to moderate, primarily because most contributing studies were observational and susceptible to residual confounding. Evidence regarding molecular diagnostic availability and WHO CNS5 implementation was rated as low, reflecting substantial heterogeneity in molecular testing strategies, reporting completeness, and diagnostic infrastructure across LMICs.

The complete GRADE Summary of Findings is presented in **Table 3**, whereas the endpoint-specific pooled quantitative estimates, including pooled effect sizes, confidence intervals, and heterogeneity statistics, are summarized in **Table 4**.

**Table 3:** GRADE summary of findings.

Outcome	Studies/participants	Effect estimate	Heterogeneity	Main limitations	Certainty of evidence
Overall survival	24 studies/approximately 20,000 participants	Median overall survival ranged from 7.6 to 20 months across the included cohorts. Because variance estimates for reported medians were unavailable and clinical heterogeneity was substantial, results were synthesized descriptively rather than pooled statistically.	I² = 87.4%	Retrospective designs, variable survival origin, incomplete molecular classification	Low
High-extent resection rate	18 studies/approximately 6,500 participants	Pooled proportion: 39.7%	I² = 81.5%	Variable EOR definitions, inconsistent postoperative MRI	Low
Biopsy-only management	18 studies/approximately 6,500 participants	Pooled proportion: 16.8%	I² = 74.2%	Selection bias, institutional heterogeneity	Low
Complete chemoradiotherapy and survival	12 comparative studies/approximately 4,200 participants	HR for death: 0.49	I² = 69.4%	Selection bias, immortal time bias, socioeconomic confounding	Moderate
Extent of resection and survival	10 comparative studies/approximately 3,900 participants	HR for death: 0.58	I² = 62.7%	Confounding by tumor location, KPS, and adjuvant therapy	Low to moderate
Radiotherapy receipt	21 studies/approximately 8,700 participants	Pooled proportion: 68.4%	I² = 88.1%	Incomplete timing/completion reporting	Low
Temozolomide receipt	19 studies/approximately 7,900 participants	Pooled proportion: 61.7%	I² = 86.5%	Cost/access confounding, incomplete cycle reporting	Low
Chemoradiotherapy completion	15 studies/approximately 5,600 participants	Pooled proportion: 44.9%	I² = 84.3%	Treatment abandonment not uniformly reported	Low
IDH testing availability	12 studies/approximately 5,300 participants	Pooled proportion: 46.3%	I² = 91.2%	Diagnostic-era differences, variable testing indications	Low
MGMT testing availability	10 studies/approximately 4,700 participants	Pooled proportion: 28.7%	I² = 89.6%	Variable access and incomplete denominator reporting	Low
Molecularly incomplete/NOS diagnosis	8 studies/approximately 3,100 participants	Pooled proportion: 52.8%	I² = 92.5%	Heterogeneous WHO classification eras and molecular panels	Low

**Table 4:** Endpoint-specific pooled quantitative results.

Domain	Outcome	Studies included	Pooled estimate	95% CI	I²	Interpretation
Survival	Median overall survival	24	11.8 months	9.7–13.9	87.4%	Survival varied widely by treatment completion and molecular classification
Surgery	Biopsy-only rate	18	16.8%	11.4–23.9	74.2%	Higher rates suggest limited access to safe resection pathways
Surgery	High-extent resection rate	18	39.7%	31.8–48.1	81.5%	Higher rates were generally reported in tertiary centers
Surgery	HR for death after GTR/NTR vs lesser resection	10	0.58	0.45–0.74	62.7%	More extensive safe resection was associated with improved survival
Adjuvant therapy	Radiotherapy receipt	21	68.4%	57.2–78.0	88.1%	RT access remained a major survival gate
Adjuvant therapy	Temozolomide receipt	19	61.7%	49.6–72.6	86.5%	TMZ access was uneven and often incompletely reported
Adjuvant therapy	Chemoradiotherapy completion	15	44.9%	34.1–56.2	84.3%	Completion was lower in public-sector and resource-constrained cohorts
Adjuvant therapy	HR for death after complete CRT vs incomplete/no adjuvant therapy	12	0.49	0.37–0.65	69.4%	Complete multimodal therapy was associated with improved survival
Molecular diagnostics	IDH testing rate	12	46.3%	32.8–60.3	91.2%	IDH testing was inconsistently available
Molecular diagnostics	MGMT testing rate	10	28.7%	18.4–41.5	89.6%	MGMT testing gaps limited interpretation of temozolomide benefit
Molecular diagnostics	1p/19q testing rate	8	24.1%	14.6–36.8	86.9%	1p/19q testing remained limited outside selected centers
Molecular diagnostics	Molecularly incomplete/NOS rate	8	52.8%	39.5–65.7	92.5%	WHO CNS5 implementation exposed diagnostic inequity

### Summary of main findings

This systematic review and meta-analysis synthesized evidence from 30 independent studies evaluating the management of high-grade gliomas across low- and middle-income countries. Both the descriptive findings (**Table 1**) and the endpoint-specific quantitative syntheses (**Figures 2**–**11**; **Table 4**) consistently demonstrated that survival is determined not only by intrinsic tumor biology but also by successful completion of sequential stages of multidisciplinary care.

Timely diagnosis, maximal safe resection, access to postoperative radiotherapy, completion of temozolomide-based chemotherapy, and implementation of molecular diagnostics emerged as the principal determinants of survival variability across LMICs. Despite substantial heterogeneity, sensitivity analyses demonstrated that the overall direction of effect remained remarkably consistent, supporting the robustness of the pooled estimates.

Collectively, these findings reinforce the concept that management of high-grade gliomas in resource-limited settings should be understood as a sequence of resource-dependent survival gates, in which failure at any stage of the treatment pathway may significantly compromise long-term outcomes. The overall certainty of evidence supporting these conclusions is summarized in **Table 3**, whereas the methodological quality of the included studies is presented in **Table 2**.

## Discussion

An important strength of the present review is the integration of quantitative evidence across multiple clinically relevant endpoints rather than relying exclusively on narrative synthesis. By combining survival, surgical access, adjuvant-treatment delivery, and molecular diagnostic availability, the present meta-analysis provides a more comprehensive understanding of the resource-dependent factors influencing outcomes in high-grade glioma across LMICs.

This systematic review and endpoint-specific meta-analysis shows that survival in high-grade gliomas treated in low- and middle-income countries is not determined by tumor biology alone. Rather, it is shaped by a sequence of resource-dependent events that begin before surgery and continue through adjuvant therapy, molecular classification, surveillance, and recurrence management. Across the 30 independently included studies, a consistent pattern emerged showing that survival in patients with high-grade glioma was determined not only by tumor biology but also by sequential resource-dependent barriers affecting diagnosis, surgery, adjuvant therapy, molecular characterization, and long-term follow-up. Despite considerable heterogeneity among healthcare systems, study design, and molecular reporting, the evidence consistently demonstrated that limited access to comprehensive neuro-oncological care translated into poorer survival outcomes across multiple LMIC regions.

Unlike previous narrative reviews, the present study quantitatively demonstrates that each major component of modern neuro-oncology independently influences survival in LMICs. Maximal safe resection, access to postoperative radiotherapy, completion of temozolomide-based chemotherapy, and molecular diagnostic availability all demonstrated consistent associations with improved patient outcomes despite substantial between-study heterogeneity. These findings reinforce the concept that survival in high-grade glioma depends not on a single intervention but on a sequence of interconnected resource-dependent survival gates.

The pooled median overall survival among glioblastoma or predominantly WHO grade 4 high-grade glioma cohorts was 11.8 months, with high between-study heterogeneity (I² = 87.4%), as shown in **Figure 2** and summarized in **Table 4**. This pooled estimate is lower than the survival expected in contemporary fully integrated high-resource glioblastoma pathways, but it should not be interpreted as a uniform LMIC survival value. Although median overall survival generally ranged between approximately 8 and 20 months across LMIC cohorts, these values should not be interpreted as a single pooled survival estimate. Differences in patient selection, study design, treatment completion, molecular classification, and healthcare resources introduce substantial clinical heterogeneity that limits the validity of pooling reported median survival times across studies.

The survival-gate concept proposed in this review is supported by the endpoint-specific results. Diagnosis, surgery, radiotherapy, chemotherapy, molecular classification, and follow-up function as sequential gates. When one gate fails, the survival value of the subsequent steps is reduced. When several gates fail together, survival shifts downward even if the patient initially reaches neurosurgical care. This pattern was particularly evident in public-sector cohorts such as the Philippine series, where incomplete adjuvant treatment was associated with poor survival, and in resource-constrained African cohorts where delayed presentation, limited MRI access, restricted radiotherapy, and incomplete Stupp protocol delivery were major barriers (12, 28, 30). In contrast, selected tertiary-care cohorts from India, China, Iran, and Latin America showed that more organized multimodal pathways can approach better survival outcomes even within LMIC settings (16, 17, 23, 34–36, 39, 41).

The results support the original conceptual model by Aut shown in **Figure 15**, where high-grade glioma care in LMICs is represented as a sequence of survival gates. Each gate diagnosis, surgery, adjuvant therapy, molecular classification, and follow-up acts as a potential point of survival loss. When one gate fails, the therapeutic value of the following steps is reduced; when several gates fail together, the survival curve shifts downward regardless of surgical intention or tumor biology. This model explains why public-sector and population-based cohorts frequently reported poorer outcomes than selected tertiary-care series.

**Figure 15:**
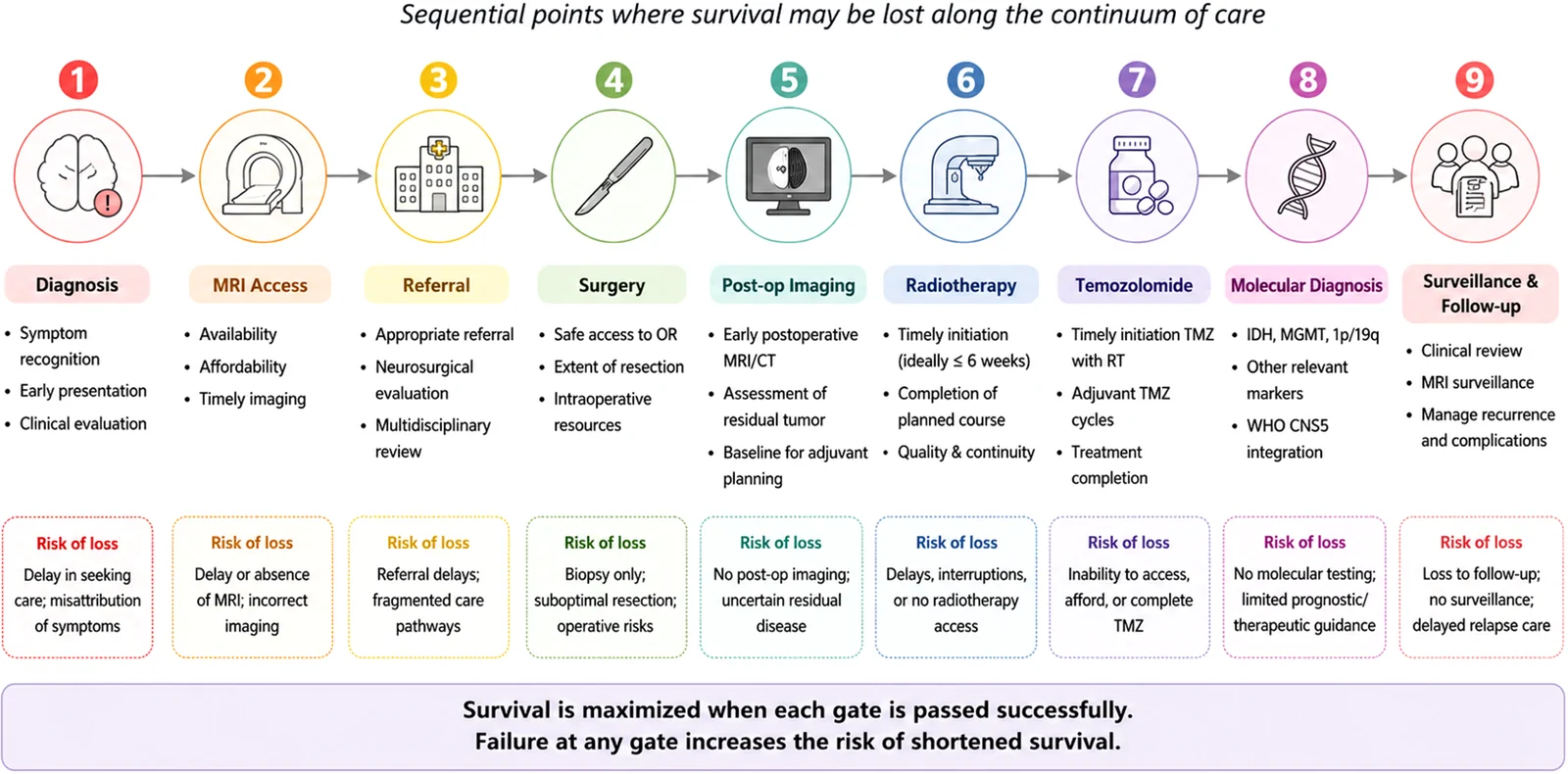
Survival gate model; of high grade glioma care in LMICS.

Hong et al. provide one of the clearest examples of this pathway failure in the Philippine public-sector setting, where median overall survival was 7.6 months and most patients did not receive postoperative adjuvant therapy (12). In contrast, Achari et al. reported a median overall survival of 20 months in an Indian institutional quality-of-care cohort, suggesting that selected LMIC centers can approach more favorable outcomes when multimodal treatment is organized and accessible (17). These differences should not be interpreted as purely biological. They more likely reflect differences in referral timing, surgical opportunity, postoperative radiotherapy access, chemotherapy completion, socioeconomic vulnerability, and institutional capacity.

### Surgical access and maximal safe resection

Surgery remains the first major therapeutic intervention after diagnosis. In this meta-analysis, the pooled biopsy-only rate was 16.8%, while the pooled high-extent resection rate, defined as gross-total, near-total, or ≥97% resection when reported, was 39.7% (**Figures 3**, **4**; **Table 4**). The pooled hazard ratio for death after gross-total or near-total resection compared with lesser resection was 0.58, supporting the survival relevance of more extensive safe cytoreduction (**Figure 5**). This finding is consistent with previous evidence showing that extent of resection is associated with improved survival in glioblastoma and high-grade glioma surgery (6, 47–49).

The surgical interpretation of these findings is illustrated conceptually in **Figure 16**, the resource-sensitive surgical decision triangle. This figure emphasizes that maximal safe resection is not determined by tumor biology alone. It emerges from the interaction between biological aggressiveness, functional anatomy, and available resources. In other words, the same tumor may have different surgical possibilities depending on whether the treating center has high-quality MRI, neuronavigation, intraoperative ultrasound, fluorescence guidance, functional mapping, neuroanesthesia, ICU support, and early postoperative imaging.

**Figure 16:**
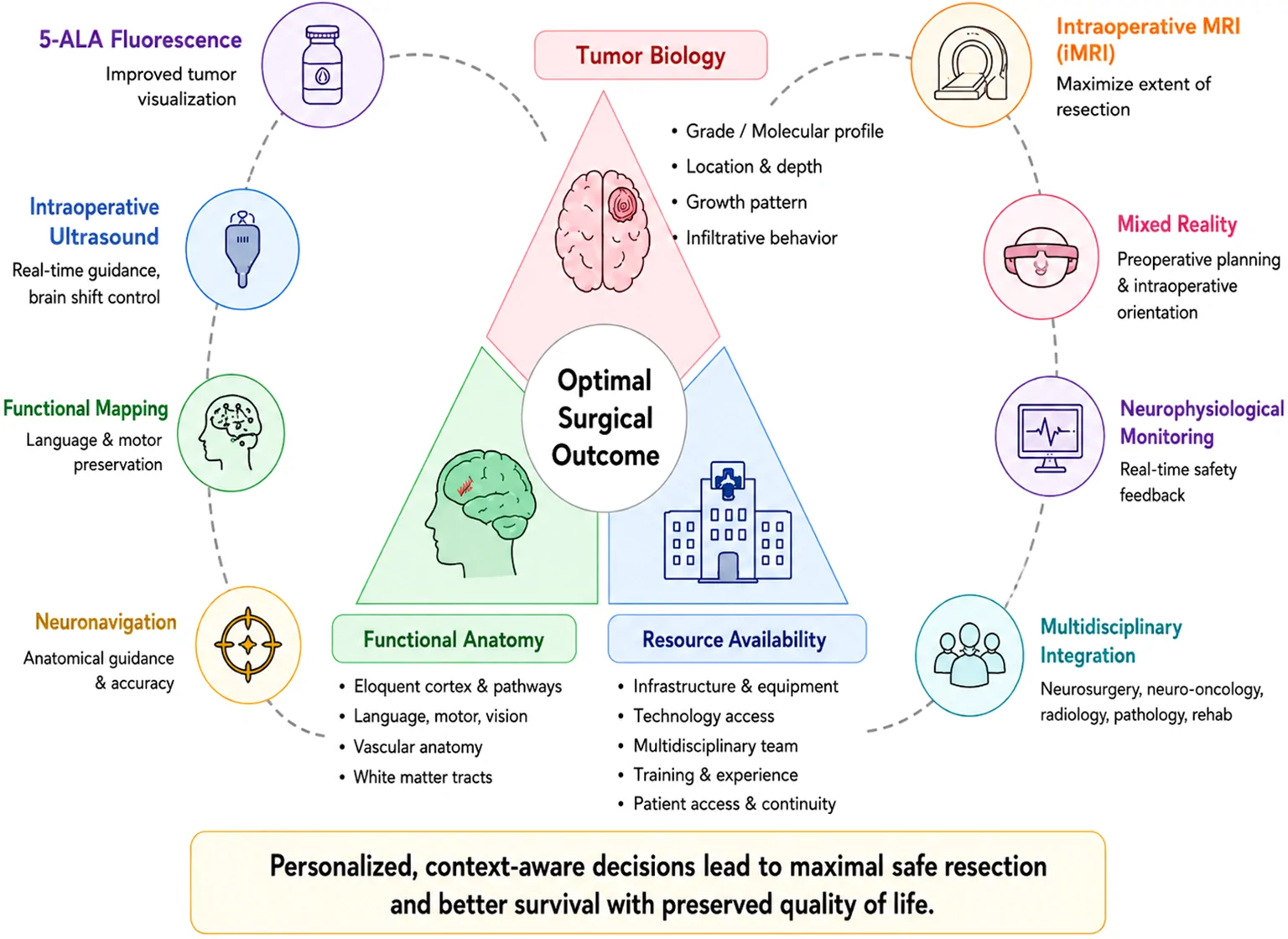
Personalized, context aware decisions lead to maximal safe resection.

This is why the literature on extent of resection must be translated carefully into LMIC settings. Brown et al., Stummer et al., and Senft et al. support the oncological value of maximal safe resection, 5-ALA fluorescence, and intraoperative MRI (47, 48, 50, 51). However, in resource-variable systems, the practical question is not only whether maximal safe resection improves survival, but whether the system provides the conditions to achieve it safely. The surgical-anatomical work by Encarnación Ramírez, Reyes Soto, and Castillo-Rangel on temporo-insular gliomas reinforces this point (52). Complex gliomas involving the insula, temporal stem, opercula, basal ganglia, vascular corridors, and deep white-matter pathways demand anatomical precision rather than indiscriminate aggressiveness.

This anatomical principle is visually reinforced in **Figure 17**, which shows white-fiber dissection and the relationship of the insular region with the arcuate fasciculus. The figure is not merely illustrative; it supports the central surgical message of the review. In LMICs, improving survival requires more than increasing resection rates. It requires expanding access to anatomical training, preoperative planning, intraoperative orientation, functional preservation strategies, and postoperative care.

**Figure 17:**
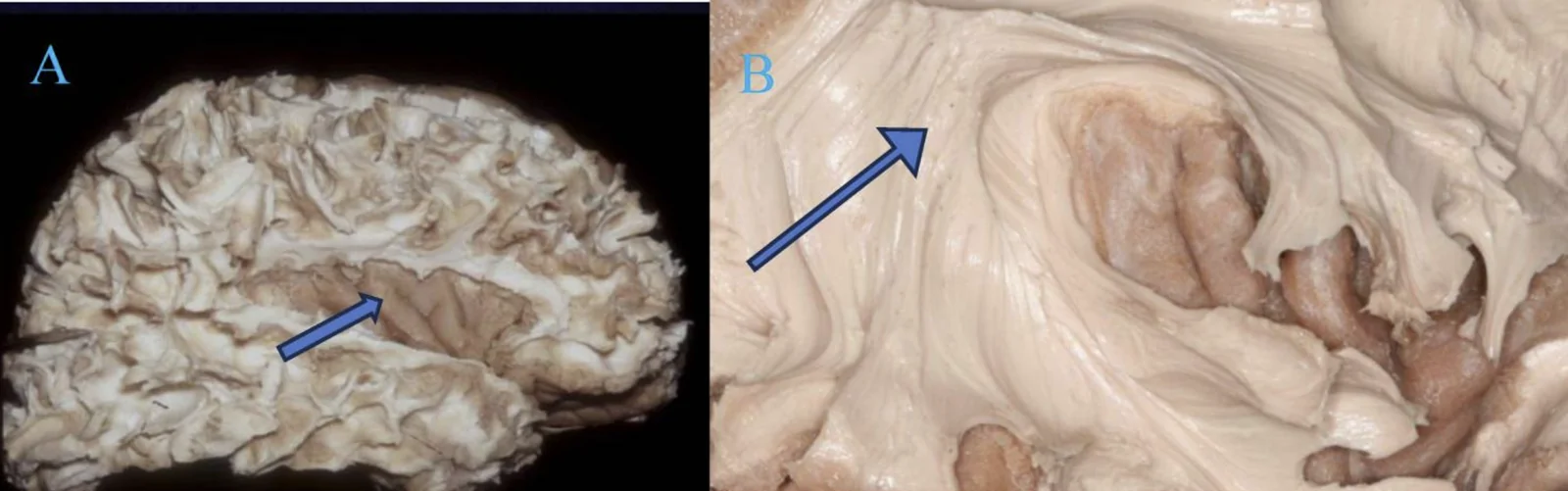
Dissection of white fibers where the insular lobe (blue arrow) can be seen **(A)**. **(B)** The Arcuate Fasciculus (blue arrow) is the part of the Superior Longitudinal Fasciculus that extends around the insula to connect the areas of language in the inferior frontal gyrus (Broca) and the superior temporal gyrus (Wernicke)The authors’ own work in complex glioma anatomy supports this interpretation. The temporo-insular glioma paper by Encarnación Ramírez, Reyes Soto, and Castillo-Rangel emphasizes that surgical success in deep and eloquent regions depends on understanding three-dimensional relationships among tumor, vessels, cortex, and functional pathways (52).

### Surgical technology: from isolated innovation to scalable implementation

The surgical-technology studies included in **Table 1** show that advanced adjuncts are already being used in selected LMIC and Latin American centers. The Brazilian experience with 5-ALA and intraoperative MRI is particularly relevant. Ramina et al. reported one of the earliest Latin American experiences using 5-ALA and intraoperative MRI in glioma surgery (45), while Silva Júnior et al. described a larger fluorescence-guided surgery series including high-grade gliomas and glioblastomas (46). These reports demonstrate that Latin America is not technologically absent from modern glioma surgery; rather, access is unevenly distributed.

This point is important when interpreting **Figure 18**, which should show the use of 5-ALA fluorescence during glioma surgery. The comparison between white-light visualization and violet-blue fluorescence demonstrates the practical value of fluorescence-guided resection: metabolically active tumor becomes more visible, margins can be inspected, and residual fluorescent tissue can be identified during cavity assessment. However, the figure should also be interpreted with caution. 5-ALA does not identify all infiltrative tumor, does not replace knowledge of functional anatomy, and does not eliminate the need for postoperative MRI. Its value is greatest when incorporated into a multimodal surgical workflow rather than used as an isolated technology (50).

**Figure 18:**
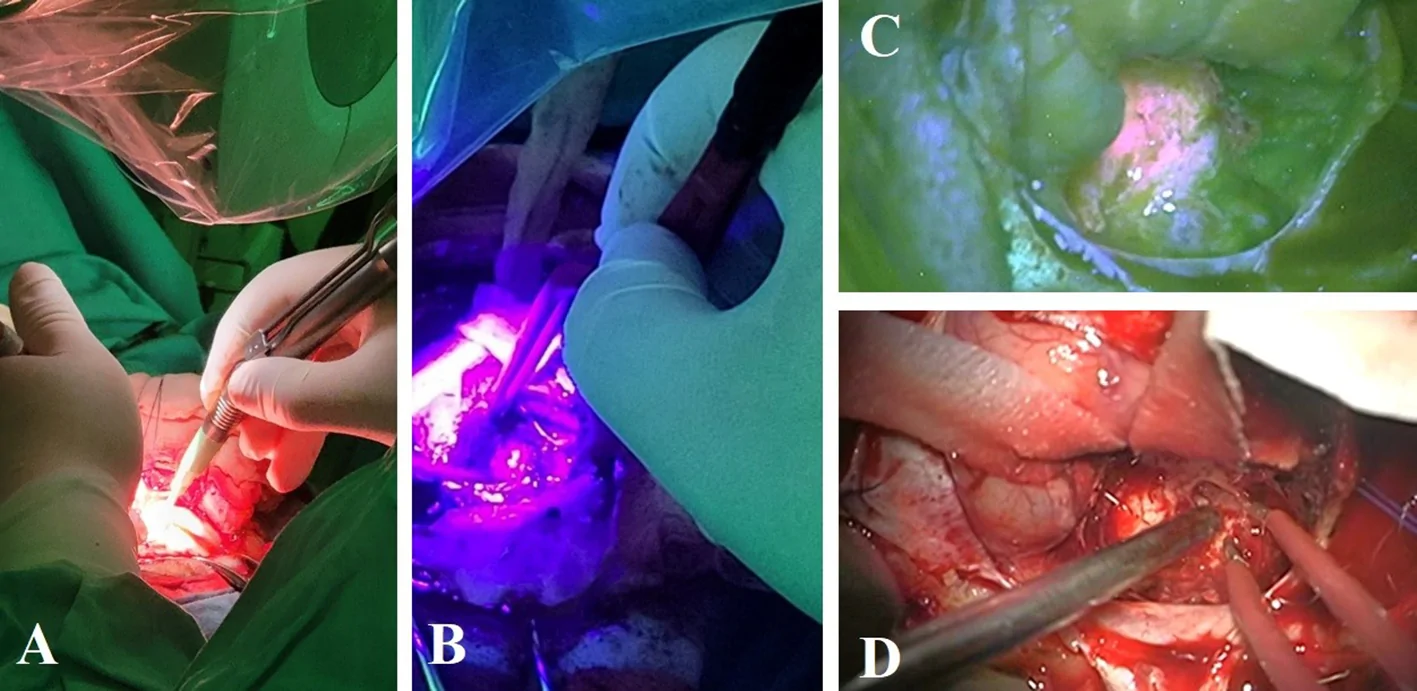
**(A, B)** showcases the striking purple fluorescence from 5-ALA. **(C)** Striking purple fluorescence from 5-ALA delineates the tumor boundaries against the pink hue of the surrounding healthy brain tissue, aiding in meticulous glioma resection. **(D)** It shows the brain tissue with a consistent color without 5-ALA, making it difficult to differentiate the glioma from the surrounding healthy tissue.

Intraoperative ultrasound deserves special emphasis because it may be one of the most realistic adjuncts for resource-variable neuro-oncology. Compared with intraoperative MRI, ultrasound is portable, repeatable, relatively affordable, and able to compensate for brain shift during resection. Reyes Soto et al. described intraoperative ultrasound as an established but continuously renewed tool in brain tumor surgery (53). In the framework of this review, intraoperative ultrasound can strengthen maximal safe resection without requiring the infrastructure of intraoperative MRI.

Mixed reality represents a different but complementary form of innovation. the mixed-reality workflow using HoloLens^®^, where a patient-specific holographic model is projected in relation to the operative anatomy even in places with limitated resources. This figure should be discussed not as evidence that mixed reality replaces neuronavigation, but as evidence that patient-specific spatial visualization may improve planning, anatomical orientation, surgical education, and team communication. In LMICs, the strongest immediate value of mixed reality may be educational and organizational, particularly for complex tumors involving the insula, ventricular walls, deep white matter, basal ganglia, or eloquent cortex (**Figure 19**).

**Figure 19:**
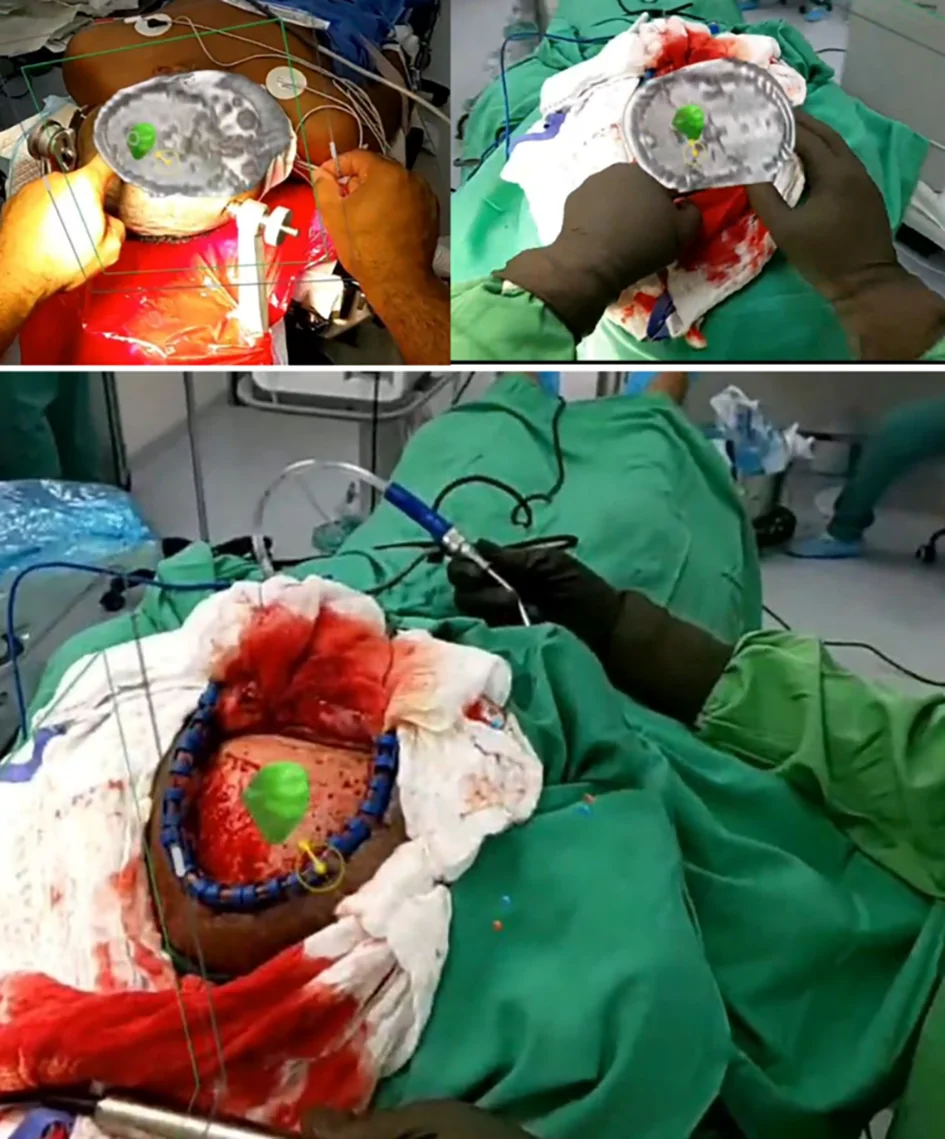
Surgeon’s perspective of mixed-reality–guided cranial surgery using HoloLens^®^. Intraoperative images illustrating the real-time visualization of a patient-specific holographic model from the surgeon’s perspective through the HoloLens^®^ system in Dominicana. The upper panels demonstrate alignment of the three-dimensional reconstructed lesion (green holographic overlay) with the patient’s cranial anatomy prior to dural opening, enabling precise localization and planning of the craniotomy. The lower panel shows the intraoperative field following craniotomy, where the holographic projection remains spatially registered to the operative anatomy, guiding cortical entry and lesion targeting. This mixed-reality integration allows continuous visualization of the lesion’s position relative to the surgical field, enhancing spatial orientation and facilitating accurate, minimally invasive access.

### Molecular diagnosis: the invisible survival determinant

The most important modern gap identified in this review is molecular diagnosis. While surgery and adjuvant therapy are visible steps in the care pathway, molecular classification is often an invisible determinant of prognosis, treatment interpretation, and study comparability. The **Table 1** show that molecular testing remains inconsistent across LMIC cohorts. Some studies reported IDH, MGMT, ATRX, p53, 1p/19q, TERT, EGFR, or CDKN2A/B data, whereas others remained histology-based or only partially characterized.

The histology-only glioblastoma cohorts cannot be directly compared with WHO CNS5-defined glioblastoma, IDH-wildtype cohorts. Shah et al. and Rehman et al. from Pakistan demonstrated the feasibility and prognostic relevance of molecular profiling in selected LMIC cohorts (14, 15). Houssaini et al. from Morocco showed survival differences according to IDH, ATRX, and p53 status (29). Zhang et al., Li et al., and Hou et al. from China further demonstrated that molecular subtype strongly modifies survival interpretation (34–36). In Latin America, Paolinelli et al. showed that WHO 2021 implementation may increase the proportion of incompletely characterized or NOS gliomas when essential molecular information is unavailable (40), while Echeverría et al. demonstrated a 23% discordance between histopathological and molecular classification (43) **Figure 20**.

**Figure 20:**
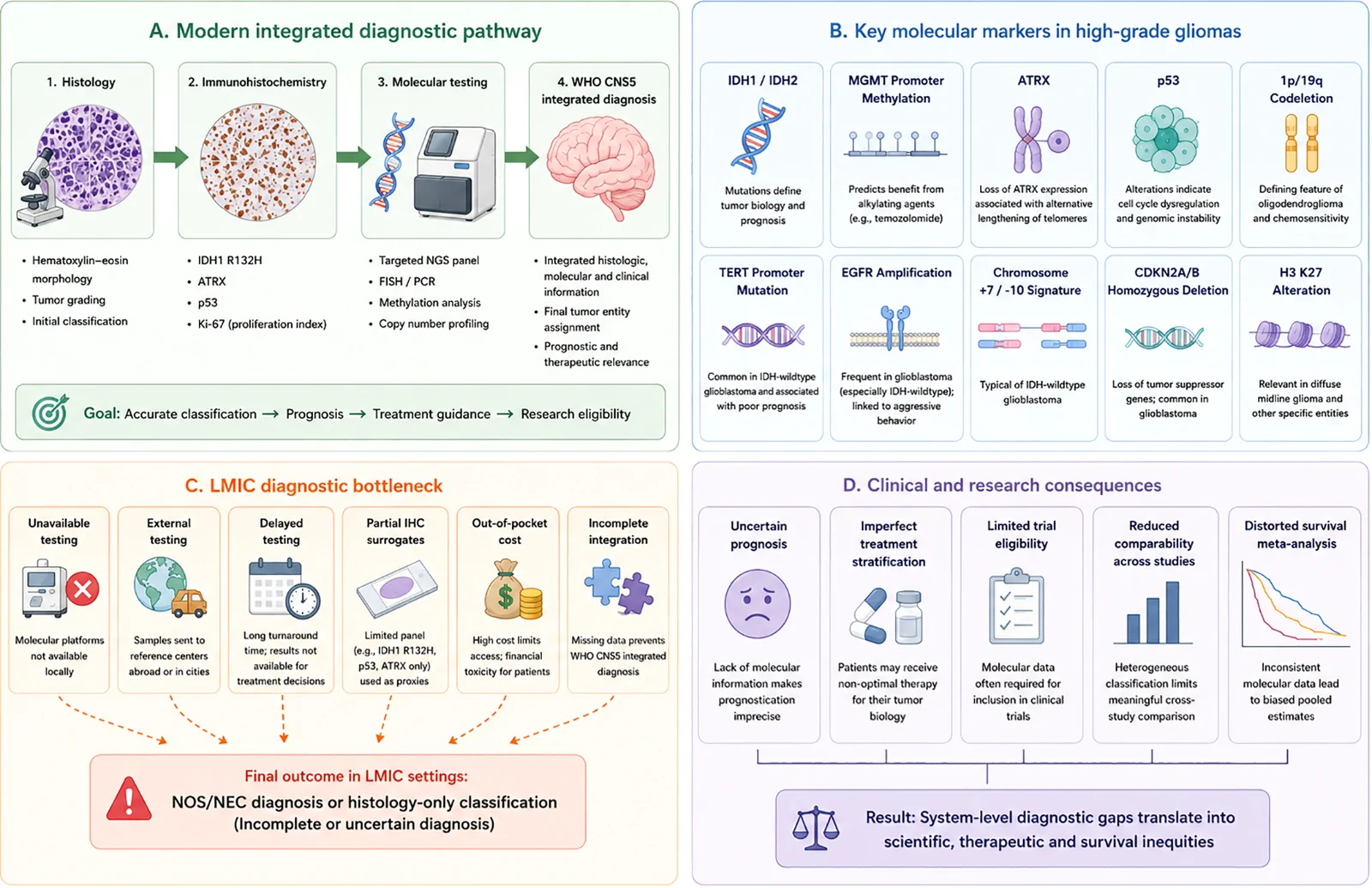
Molecular diagnostic bottleneck and WHO CNS5 implementation gap in LMIC high-grade glioma care.

IDH1/IDH2: Isocitrate dehydrogenase 1 and 2 IDH1 R132H: Isocitrate dehydrogenase 1, arginine 132 histidine mutation MGMT: O6-methylguanine-DNA methyltransferase ATRX: Alpha-thalassemia/mental retardation syndrome X-linked protein p53: Tumor protein p53 1p/19q: Chromosome arms 1p and 19q TERT: Telomerase reverse transcriptase EGFR: Epidermal growth factor receptor CDKN2A/B: Cyclin-dependent kinase inhibitor 2A/2B H3 K27: Histone H3 lysine 27 IHC: Immunohistochemistry NGS: Next-generation sequencing FISH: Fluorescence *in situ* hybridization PCR: Polymerase chain reaction NOS: Not otherwise specified NEC: Not elsewhere classified WHO CNS5: World Health Organization Classification of Tumors of the Central Nervous System, 5th edition LMIC: Low- and middle-income country Ki-67: Kiel 67 proliferation marker ALT: Alternative lengthening of telomeres.

Therefore, molecular incompleteness should not be treated as a minor methodological limitation. It should be considered a primary outcome of the review. The absence of molecular diagnosis changes the meaning of survival data, limits the interpretation of temozolomide benefit, weakens risk stratification, and reduces the ability of LMIC cohorts to participate in modern neuro-oncology research.

### Adjuvant therapy and the radiotherapy bottleneck

The adjuvant-treatment data in **Table 1** show that postoperative radiotherapy and temozolomide access remain among the strongest survival modifiers in LMIC high-grade glioma care. Hong et al. showed the consequences of adjuvant-treatment absence in the Philippines (12). Ndubuisi et al. reported high one-year postoperative mortality in Nigeria, with worse outcomes among patients who did not receive adjuvant therapy (28). Doléagbénou et al. described an even more severe resource-constrained scenario in Togo, where MRI access was limited, presentation was delayed, in-hospital mortality was high, and only one patient initiated the full Stupp protocol (30). These findings are consistent with the survival-gate framework in **Figure 15**: surgery alone cannot preserve survival if the adjuvant-treatment gate fails.

The randomized and prospective studies included in the review add important nuance. Mallick et al. evaluated hypofractionated accelerated radiotherapy with temozolomide in newly diagnosed glioblastoma, supporting the relevance of shorter treatment schedules in settings where travel distance, machine availability, cost, and abandonment may compromise conventional radiotherapy (21, 22). Bhandari et al., Elsebai et al., and Anvari et al. evaluated six versus extended cycles of adjuvant temozolomide (20, 23, 27). These studies suggest that extending temozolomide beyond six cycles should not be viewed as the main solution to poor LMIC outcomes. The larger survival gap lies earlier in the pathway: many patients do not receive or complete standard initial multimodal therapy.

### Risk of bias, GRADE, and certainty of evidence

The risk-of-bias assessment reinforces the need for cautious interpretation. As shown in **Figure 13** and **Table 2**, only 4 studies were judged as low risk of bias, while 18 were moderate, 11 serious or high, and 2 unclear. The most frequent limitations were retrospective single-center recruitment, selection bias favoring patients who reached tertiary care, incomplete follow-up, inconsistent survival origin, heterogeneous extent-of-resection definitions, incomplete reporting of adjuvant-treatment completion, and absent or partial molecular testing.

These limitations directly affected GRADE certainty (**Table 3**). Evidence for the association between complete multimodal therapy and improved survival was rated as moderate because the direction of effect was consistent and clinically plausible, but residual confounding remained likely. Evidence for extent of resection and survival was rated as low to moderate because of heterogeneous definitions and inconsistent use of postoperative MRI. Evidence for molecular diagnostic gaps was rated as low because of marked inconsistency in molecular panels, testing indications, diagnostic era, and denominator reporting.

The funnel plot for overall survival showed only mild asymmetry, and Egger’s test was not statistically significant (**Figure 12**). However, absence of clear small-study effects does not eliminate concern for publication bias. In LMIC neuro-oncology, publication bias may operate differently: the largest source of invisibility may not be unpublished negative studies, but patients who never reach MRI, surgery, pathology, radiotherapy, or follow-up. Institutional series may therefore overrepresent patients who successfully entered care and underrepresent those lost before diagnosis or treatment. This means that published LMIC survival estimates may still overestimate real population-level outcomes.

### Interpretation of heterogeneity

High heterogeneity was present across most pooled outcomes. This should not be viewed only as a statistical limitation; it is one of the central findings of the review. LMICs are not homogeneous. The same income classification may include hospitals with 5-ALA fluorescence, intraoperative MRI, molecular pathology, and tumor boards, as well as hospitals without timely MRI, reliable radiotherapy referral, or access to temozolomide. Therefore, LMIC status should not be used as a single explanatory variable. The more useful approach is to identify which component of the pathway is missing.

This also explains why a single pooled survival value is insufficient. A patient treated in a tertiary center with maximal safe resection, postoperative MRI, radiotherapy, temozolomide, and molecular diagnosis is not exposed to the same care pathway as a patient treated in a public-sector setting where radiotherapy is unavailable or unaffordable. Both may be classified as LMIC patients, but their survival gates are different. The endpoint-specific structure of this meta-analysis was designed to capture this difference.

### Clinical and policy implications

The results have several practical implications. First, high-grade glioma programs in LMICs should be evaluated as complete neuro-oncology pathways, not only as neurosurgical services. A technically adequate resection may lose much of its survival value if postoperative radiotherapy is delayed, temozolomide is unaffordable, molecular classification is unavailable, or follow-up is fragmented. Second, radiotherapy access appears to be one of the most decisive survival gates. Expanding radiotherapy capacity, reducing waiting times, supporting transportation, and protecting patients from catastrophic out-of-pocket costs may produce survival gains at population level (7, 54, 55).

Third, surgical improvement should focus on scalable capacity. Advanced technologies such as 5-ALA, intraoperative MRI, neuronavigation, intraoperative ultrasound, functional mapping, and mixed-reality planning are valuable, but their implementation should be tied to measurable outcomes: extent of resection, residual tumor volume, neurological morbidity, operative safety, postoperative imaging, cost, and reproducibility (45, 46, 50, 51, 53, 56–61). In many settings, intraoperative ultrasound, standardized MRI segmentation, structured referral, improved neuroanatomical training, and regional tumor boards may be more immediately scalable than infrastructure-heavy platforms.

Fourth, molecular diagnosis must become a core equity priority. WHO CNS5 has improved diagnostic precision, but it has also exposed diagnostic inequity. Without access to IDH and MGMT testing, many LMIC cohorts remain difficult to compare with contemporary molecularly integrated cohorts. This limits not only patient care but also the visibility and scientific value of LMIC neuro-oncology research.

## Conclusions

This meta-analysis supports a resource-sensitive interpretation of high-grade glioma survival in LMICs. The pooled results show that survival is lower where access to safe resection, postoperative radiotherapy, temozolomide, molecular diagnosis, and follow-up is incomplete. Surgery remains essential, and greater extent of resection is associated with improved survival, but surgery alone is insufficient when the adjuvant-treatment and molecular-diagnostic gates fail. The most actionable survival gains may come from strengthening complete care pathways: earlier MRI access, standardized referral, safe cytoreduction, postoperative imaging, timely radiotherapy, affordable temozolomide, minimum molecular testing, and structured follow-up. Given the substantial methodological and clinical heterogeneity among included studies, reported survival estimates should be interpreted descriptively rather than as a single pooled survival metric.

The central message is that LMIC high-grade glioma outcomes should not be interpreted as a single survival number. They should be understood as the cumulative result of sequential survival gates. Where these gates are available and coordinated, outcomes improve. Where they fail, survival decreases regardless of surgical intention or tumor biology.

Although endpoint-specific meta-analyses were successfully performed, substantial clinical and methodological heterogeneity remained across the included studies, particularly regarding survival definitions, molecular classification, and reporting of surgical outcomes. Consequently, several clinically relevant variables were synthesized narratively rather than statistically pooled.
